# The Delphi Delirium Management Algorithms. A practical tool for clinicians, the result of a modified Delphi expert consensus approach

**DOI:** 10.56392/001c.90652

**Published:** 2024-01-12

**Authors:** Thomas H. Ottens, Carsten Hermes, Valerie Page, Mark Oldham, Rakesh Arora, O. Joseph Bienvenu, Mark van den Boogaard, Gideon Caplan, John W. Devlin, Michaela-Elena Friedrich, Willem A. van Gool, James Hanison, Hans-Christian Hansen, Sharon K. Inouye, Barbara Kamholz, Katarzyna Kotfis, Matthew B. Maas, Alasdair M.J. MacLullich, Edward R. Marcantonio, Alessandro Morandi, Barbara C. van Munster, Ursula Müller-Werdan, Alessandra Negro, Karin J. Neufeld, Peter Nydahl, Esther S. Oh, Pratik Pandharipande, Finn M. Radtke, Sylvie De Raedt, Lisa J. Rosenthal, Robert Sanders, Claudia D. Spies, Emma R.L.C. Vardy, Eelco F. Wijdicks, Arjen J.C. Slooter

**Affiliations:** 1 Intensive Care Unit, Haga Teaching Hospital,; 2 Intensive Care Medicine, University Medical Center Utrecht,; 3 Critical Care, Watford General Hospital,; 4 School of Medicine and Dentisty, University of Rochester,; 5 Psychiatry, University of Rochester Medical Center,; 6 Harrington Heart and Vascular Institute, University Hospitals of Cleveland,; 7 Division of Cardiac Surgery, Case Western Reserve University,; 8 School of Medicine, Johns Hopkins University,; 9 Intensive Care Medicine, Radboud University Nijmegen Medical Centre,; 10 School of Clinical Medicine, UNSW Sydney,; 11 Geriatric Medicine, Prince of Wales Hospital,; 12 Division of Pulmonary and Critical Care Medicine, Brigham and Women’s Hospital,; 13 Bouve College of Health Sciences, Northeastern University,; 14 Abteilung für Sozialpsychiatrie, Hollabrunn, Austria,; 15 Neurology, Amsterdam University Medical Centers,; 16 Anaesthesia, Manchester University NHS Foundation Trust,; 17 Neurology, Friedrich-Ebert-Krankenhaus,; 18 Beth Israel Deaconess Medical Center,; 19 Harvard Medical School,; 20 Anaesthesia, Intensive Care and Acute Poisoning, Pomeranian Medical University,; 21 Neurology, Northwestern Medicine,; 22 Feinberg School of Medicine, Northwestern University,; 23 Usher Institute Ageing and Health, University of Edinburgh,; 24 Geriatric Medicine, Beth Israel Deaconess Medical Center,; 25 Rehabilitation, Fondazione Teresa Camplani,; 26 Geriatric Medicine, University Medical Center Groningen,; 27 Geriatrics, Charité - Universitätsmedizin Berlin,; 28 Intensive Care Unit, IRCCS Ospedale San Raffaele,; 29 Faculty of Health Sciences, McMaster University,; 30 Intensive Care Unit, University Hospital Schleswig-Holstein,; 31 Geriatric Medicine and Gerontology, Johns Hopkins Medicine,; 32 Anesthesiology and Critical Care Medicine, Vanderbilt University Medical Center,; 33 University of Southern Denmark,; 34 Anaesthesia and Intensive Care, Nykøbing F. Hospital,; 35 Vrije Universiteit Brussel,; 36 Neurology, Universitair Ziekenhuis Brussel,; 37 Psychiatry, Northwestern Memorial Hospital,; 38 Faculty of Medicine and Health, University of Sydney,; 39 Anaesthesiology and Intensive Care, Charité - Universitätsmedizin Berlin,; 40 Northern Care Alliance NHS Foundation Trust, Oldham, United Kingdom,; 41 University of Manchester, Manchester, United Kingdom,; 42 Neurology, Mayo Clinic,; 43 Psychiatry, University Medical Center Utrecht,; 44 Brain Center, University Medical Center Utrecht

**Keywords:** Delirium, Clinical practice algorithm, Expert consensus, intensive care, cardiac surgery, hospital medicine

## Abstract

Delirium is common in hospitalised patients, and there is currently no specific treatment. Identifying and treating underlying somatic causes of delirium is the first priority once delirium is diagnosed. Several international guidelines provide clinicians with an evidence-based approach to screening, diagnosis and symptomatic treatment. However, current guidelines do not offer a structured approach to identification of underlying causes.

A panel of 37 internationally recognised delirium experts from diverse medical backgrounds worked together in a modified Delphi approach via an online platform. Consensus was reached after five voting rounds. The final product of this project is a set of three delirium management algorithms (the Delirium Delphi Algorithms), one for ward patients, one for patients after cardiac surgery and one for patients in the intensive care unit.

## INTRODUCTION

Delirium is an acute disorder of brain function that is caused by other medical conditions, substance intoxication or withdrawal, or exposure to a toxin. It is characterised by a disturbance in attention, a reduced level of orientation to the environment and other cognitive disturbances that cannot otherwise be explained by neurocognitive disorders.^1^ Delirium can be regarded as a clinical expression of acute encephalopathy.^2^ Delirium frequently occurs in hospitalised adults, and is associated with significantly increased ICU and hospital length of stay, mortality, as well as an increased risk of long-term cognitive disorders and loss of independence.^3^ The economic impact of delirium is substantial. Delirium in older hospitalised adults has been estimated to cost between $38 billion and $152 billion per year.^4^

For healthcare providers, the large variety in clinical phenotypes and fluctuating clinical course of the syndrome makes screening for and diagnosing delirium notoriously difficult. Furthermore, there is no curative treatment, and the efficacy of both pharmacologic and non-pharmacologic interventions to suppress delirium symptoms is limited.^5^ Healthcare providers have several practice guidelines, including those published by the American Geriatrics Society and the Society of Critical Care Medicine (SCCM).^5,6^ There are also guidelines for specific patient groups. Examples include the guideline for postoperative delirium from the European Society of Anaesthesiology.^7^ Although these guidelines all clearly state how and when patients should be screened for signs and symptoms of delirium, and what potential underlying causes of delirium are, none of the guidelines provides a framework for a structured approach to detection and management of underlying causes or follow-up once delirium is diagnosed.

There are several mnemonics and acronyms that aim to support healthcare providers in detecting the underlying cause of delirium. Examples include “I WATCH DEATH” and “DELIRIOUS”. However, mnemonics and acronyms often do not distinguish for what population they are intended, when and how to use the information they provide, and who developed the mnemonic.(ICU Delirium.Org, retrieved Aug 4, 2023) They also make no distinction between rare and common underlying causes of delirium, or suggest what priority should be given to the individual items.

The prevention, monitoring and treatment of delirium receives an increasing amount of attention. This is due to several factors, including evidence-based practice initiatives like SCCM’s ICU Liberation Project, as well as the arrival of simplified electroencephalography (EEG) devices that can assist in detecting the underlying EEG changes that are seen in acute encephalopathies underlying delirium.^8,9^ These innovations increase the demand for a structured framework on how to approach patients who are diagnosed with possible delirium.

This paper describes an initiative to create a clinical algorithm to provide healthcare providers with a structured approach to hospitalised patients who develop delirium.

## METHODS

In December 2020, the initiators of this project (TO, CH and AJCS) convened for the first time to discuss the existing gap in guidance for healthcare providers caring for hospitalised patients with delirium. We outlined a simple, stepwise “template algorithm” that emphasised a structured approach to detecting underlying causes of delirium, followed by suggestions for symptomatic treatments and follow-up. Model content was based on international delirium guidelines^5,7,8^ systematic reviews on underlying causes and triggers of delirium^10,11^ and mnemonics found in diverse sources (see table 1).^12^ We followed a modified Delphi approach to reach expert consensus on the contents of this model, referred to as “the Delirium Delphi Algorithms”.

With attention to diversity in training, gender and nationality, we invited a group of experts to form the interdisciplinary consensus panel. We invited 38 experts to participate. Because of the COVID-19 pandemic, the consensus process took place online. Panel members were presented with concept versions of the algorithms and were asked to respond to design elements (such as the order in which suggestions were presented), text statements (such as: *All patients should receive preventive non-pharmacologic measures, regardless of their cognitive state*) and could comment on specific textual content. Responses were gathered dichotomously (agree/disagree) and in full-text comments. The online consensus process allowed the Panel members to comment and vote privately, at their own pace.

After each voting round, the responses and comments of the Panel were discussed within the Board. The agreement between Panel members with each design choice, statement or other item offered for voting was calculated by dividing the number of members who agreed by the number of respondents to that particular voting round. Before the start of data collection, we agreed that 85% of agreement needed to be reached within the panel before a statement or design element would be consolidated in the algorithm. Elements or statements with a lower agreement level were adjusted using comments from panel members. The updated version of the algorithm was then offered to the Panel in a new voting round. Items that already reached a high level of agreement were not offered for voting again.

A report with the agreement levels and a motivation for each proposed adjustment to the algorithms was sent to the Panel members before the next voting round.

## RESULTS

In total, 38 experts agreed to participate; 37 completed at least one voting assignment (response rate 97%). TO, CH and AJCS acted as a board. Panel members had a background in anaesthesiology (5), cardiac surgery (1), geriatrics (9), intensive care medicine (7), neurology (5), nursing (4), pharmacy (1) and psychiatry and psychology (6) and practice their profession in the United States of America, Australia, Austria, Belgium, Canada, Denmark, Germany, Italy, The Netherlands, Poland, and the United Kingdom (Supplementary Material). All panel members signed the digital participation agreement before completing the first voting assignment. The voting rounds took place between June 2021 and January 2023. The panel required five voting rounds to reach consensus about the set of algorithms (Table 2).

The final product of this process is a set of three algorithms: one for patients in hospital wards, one for patients after cardiac surgery, and one for patients in intensive care units. To improve usability, the content of the algorithms was kept concise. The algorithms share a set of five “reference cards”, which contain more detailed information, such as suggestions for non-pharmacologic interventions. The full set of three algorithms and 5 reference cards, as well as an instruction for users, is presented in the Additional File 2. As an example, the algorithm version for patients in normal hospital wards is shown in Figure 1a. The reference card for non-pharmacologic interventions is presented in Figure 1b.

A detailed record of the modified Delphi voting process is presented in Additional File 1.

## DISCUSSION

Using a modified Delphi approach, we developed a set of algorithms to support healthcare providers who take care of hospitalised adult patients with delirium. In five online voting rounds, consensus was reached between a diverse group of 37 internationally recognised delirium experts.

### POSITION RELATIVE TO GUIDELINES

The Delirium Delphi Algorithms intend to provide structure and prioritisation, and strongly focus on identifying modifiable precipitating factors. As such, these are intended to be used as a tool, in addition to national and international guidelines. The content is primarily based on expert opinion and should be regarded as such. Users are advised to take their local situation into account and always comply with local legislation, particularly when it comes to involuntary medical treatments such as the use of restraints.

### KNOWLEDGE GAP

There is currently no high-quality evidence available to support one particular approach to management of delirium and its underlying causes. The application of the Delirium Delphi Algorithms implies a bundle of several “good clinical practices”. These include structured application of non-pharmacologic interventions to prevent delirium, a strong focus on the identification of modifiable precipitating factors, and follow-up that consists of frequent reassessment of the patient’s need for symptomatic drug treatments. Bundles of interventions have repeatedly been proven to be more effective in reducing the delirium burden than standalone interventions.^13^

### FUTURE OF DELIRIUM MANAGEMENT

With the arrival of innovations in delirium screening and monitoring as well as individualised interventional treatments, the field of delirium management is likely to change significantly in the next ten years. The algorithms will be kept up to date by the authors to follow these developments. We encourage healthcare providers who regularly treat patients with delirium to form interdisciplinary working groups in their institution to keep their delirium management strategies up-to-date.

## CONCLUSION

We present a set of algorithms to support healthcare providers caring for hospitalised adult patients with delirium.

## Supplementary Material

Additional File 1

Additional File 2

## Figures and Tables

**Figure 1a.**
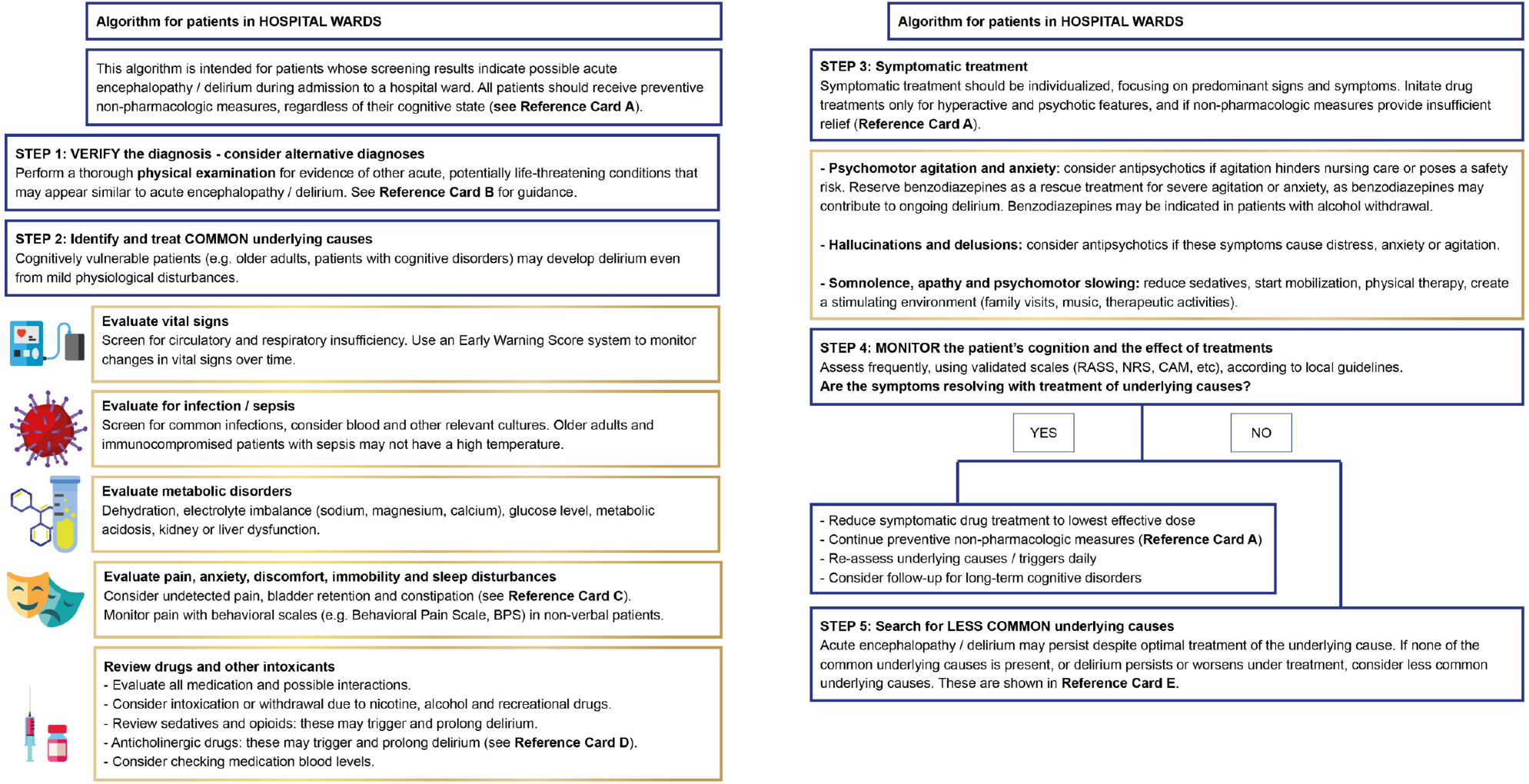


**Figure 1b.**
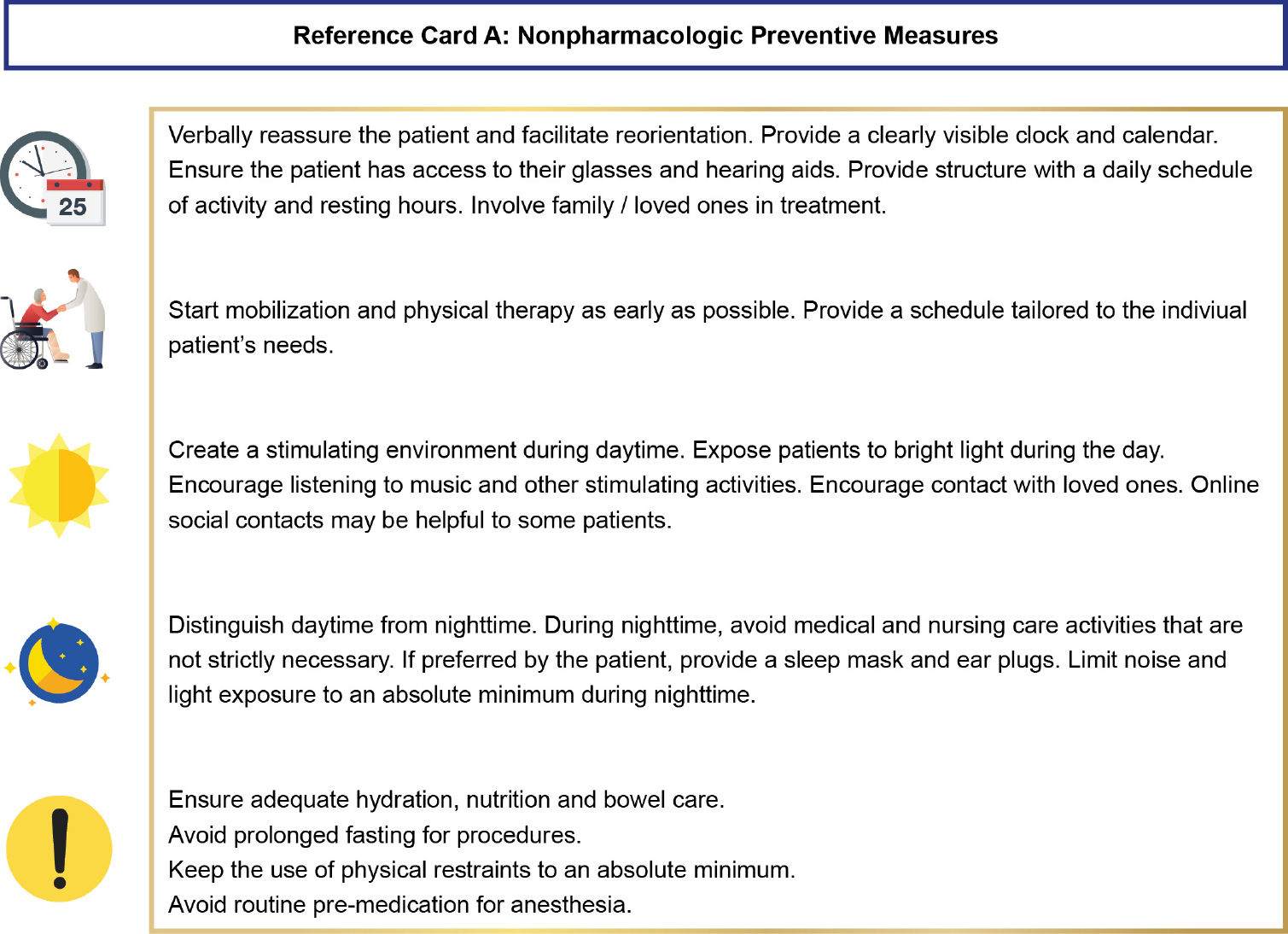


**Table 1.** Five well-known mnemonics for potential underlying causes of delirium.

“I WATCH DEATH”		“DELIRIOUS”		“DELIRIUM(S)”	
Cue	Meaning	Cue	Meaning	Cue	Meaning
**I**	**I**nfection (HIV, sepsis, pneumonia)	D	**D**rugs (Continuous drips, Na+, Ca+, BUN/ Creatinine, NH3+)	D	**D**rugs
**W**	**W**ithdrawal (alcohol, barbiturate, sedative-hypnotic)	E	**E**nvironmental factors (hearing aids, eye glasses, sleep/wake cycle)	E	**E**yes, ears, and other sensory deficits
**A**	**A**cute metabolic (acidosis, alkalosis, electrolyte disturbance, hepatic failure, renal failure)	L	**L**abs (including Na+, K+, Ca+, BUN/Cr, NH3+)	L	**L**ow O2 states (e.g. heart attack, stroke, and pulmonary embolism)
**T**	**T**rauma (closed-head injury, heat stroke, postoperative, severe burns)	I	**I**nfection	I	**I**nfection
**C**	**C**NS pathology (Abscess, hemorrhage, hydrocephalus, subdural hematoma, infection, seizures, stroke, tumors, metastases, vasculitis, encephalitis, meningitis, syphilis)	R	**R**espiratory status (ABGs-PaO2 and PCO2)	R	**R**etention (of urine or stool)
**H**	**H**ypoxia (anemia, carbon monoxide poisoning, hypotension, pulmonary or cardiac failure	I	**I**mmobility	I	**I**ctal state
**D**	**D**eficiencies (vitamin B12, folate, niacin, thiamine)	O	**O**rgan failure (renal failure, liver failure, heart failure)	U	**U**nderhydration/undernutrition
**E**	**E**ndocrinopathies (hyper/hypoadrenocorticism, hyper/ hypoglycemia, myxedema, hyperparathyroidism)	U	**U**nrecognised dementia	M	**M**etabolic causes (Diabetes, postoperative state, sodium abnormalities)
**A**	**A**cute vascular (Hypertensive encephalopathy, stroke, arrhythmia, shock)	S	**S**hock (sepsis, cardiogenic)/steroid	(S)	**S**ubdural hematoma
**T**	**T**oxins or drugs (prescription drugs, illicit drugs, pesticides, solvents)				
**H**	**H**eavy metals (lead, manganese, mercury)				

**“THINK”**		**“DR. DRE”**			
**Cue**	Meaning	Cue	Meaning		
**T**	**T**oxic Situations: CHF, shock, dehydration, delirogenic medication, new organ failure (liver, kidney)	Dr.	**D**iseases (Sepsis, COPD, CHF)		
**H**	**H**ypoxemia	Dr	**D**rug removal (spontaneous awakening trials, stopping benzodiazepines/ narcotics)		
**I**	**I**nfection/sepsis (nosocomial), Immobilization	E	**E**nvironment (Immobilisation, sleep and day/night, hearing aids, glasses)		
**N**	**N**on-pharmacological interventions Hearing aids, glasses, re-orient, sleep protocols, music, noise control, ambulation				
**K**	**K**+, Electrolyte problems				

Abbreviations: ABG, Arterial Blood Gas; BUN, Blood Urea Nitrogen; CHF, Congestive Heart Failure; CNS, Central Nervous System; HIV, Humane Immunodeficiency Virus;

**Table 2.** Voting Rounds and Response Rates

Voting round	Topic	Response Rate
**1**	Statements and structure	35/37 (94.6%)
**2**	Blueprint algorithm (ward patients) and first reference cards	35/37 (94.6%)
**3**	Improved version of blueprint algorithm and reference cards	32/37 (86.5%)
**4**	Algorithm versions for cardiac surgery and ICU population	29/37 (78.4%)
**5**	Final algorithm set, endorsement	37/37 (100%)
