# Supplementary material for: The Delphi Delirium Management Algorithms. A practical tool for clinicians, the result of a modified Delphi expert consensus approach
Source: Delirium (Bielef). Author manuscript; Available in PMC 2024 Feb 12. (PMC10861222; doi:10.56392/001c.90652)
Supplement: Additional File 1 [file NIHMS1959412-supplement-Additional_File_1.pdf]

1. **List of Panel Members**

**Name**	**Background**	**Institution**
**Board**		
Arjen Slooter	Intensivist	UMC Utrecht, Utrecht, The Netherlands
Carsten Hermes	Nurse	Bonn, Germany
Thomas Ottens	Intensivist	Hagaziekenhuis, The Hague, and UMC Utrecht, Utrecht, The Netherlands
**Members**		
Mark van den Boogaard	Nurse	Radboud University Medical Center, Nijmegen, The Netherlands
Peter Nydahl	Nurse	Universitätsklinikum Schleswig-Holstein, Kiel, Germany
Alessandra Negro	Nurse	IRCCS San Raffaele Scientific Institute, Milan, Italy
Sylvie de Raedt	Neurologist	UZ Brussel, Brussels, Belgium
Willem van Gool	Neurologist	Amsterdam University Medical Centers, Amsterdam, The Netherlands
Matthew Maas	Neurologist	Northwestern Universtity, Chicago, IL, USA
Eelco Wijdicks	Neurologist	Mayo Clinics, Rochester, MN, USA
Hans-Christian Hansen	Neurologist	Friedrich-Ebert-Krankenhaus, Germany
12 Michaela Friedrich	Psychiatrist	Klinik Floridsdorf, Vienna, Austria
13 Karin Neufeld	Psychiatrist	McMaster University, Hamilton, Canada
14 Barbara Kamholtz	Psychologist	Boston, MA, USA
15 Joe Bienvenu	Psychiatrist	Johns Hopkins, Baltimore, ML, USA
16 Lisa Rosenthal	Psychiatrist	Northwestern University, Chicago, IL, USA
17 Mark Oldham	Psychiatrist	University of Rochester MC, Rochester, NY, USA
18 Finn Radkte	Anesthesiologist	University of South Denmark, Odense, Denmark
19 Katarzyna Kotfis	Anesthesiologist	Pomeranian University, Szczecin, Poland
20 Robert Sanders	Anesthesiologist	University of Sydney, Australia
21 Claudia Spies	Anesthesiologist	Charité Universitätsmedizin, Berlin, Germany
22 Michael Avidan[1]	Anesthesiologist	Washington University, St Louis, MO, USA
23 James Hanison	Intensivist	Central Manchester University Hospitals, Manchester, UK
24 Valerie Page	Intensivist	West Hertfordshire Hospitals, UK
25 Babar Khan	Intensivist	Indiana University, IN, Indianapolis, USA
26 Wes Ely	Intensivist	Vanderbilt, Nashville, TN, USA
27 Pratik Pandharipande	Intensivist	Vanderbilt, Nashville, TN, USA
28 Alasdair MacLullich	Geriatrician	University of Edinburgh, UK
29 Allesandro Morandi	Geriatrician	Fondazione Teresa Camplani, Cremona, Italy
30 Gideon Caplan	Geriatrician	UNSW, Sydney, Australia
31 Esther Oh	Geriatrician	Johns Hopkins, Baltimore, ML, USA
32 Emma Vardy	Geriatrician	Salford Royal NHS, UK
33 Edward Marcantonio	Geriatrician	BIDMC, Boston, MA, USA
34 Barbara van Munster	Geriatrician	University Medical Center Groningen, Groningen, The Netherlands
35 Ursula Müller-Werdan	Geriatrician	Charité Universitätsmedizin, Berlin, Germany
36 Sharon Inouye	Geriatrician	BIDMC, Boston, MA, USA
37 John Devlin	Pharmacist	Northeastern, Boston, MA, USA
38 Rakesh Arora	Cardiac surgeron	University Hospitals, Cleveland, OH, USA
1. This panel member withdrew participation		

2. Proceedings from Delphi Process

Please note that the number of participants mentioned in the proceedings had to be adjusted after one of the panel members withdrew in a later phase. The documents mention 38 panel members, after the withdrawal of one panel member (Dr. Avidan), we re-calculated the agreement data before drafting the manuscript.

International Expert Consensus Panel

on the management of underlying causes of acute encephalopathy and delirium

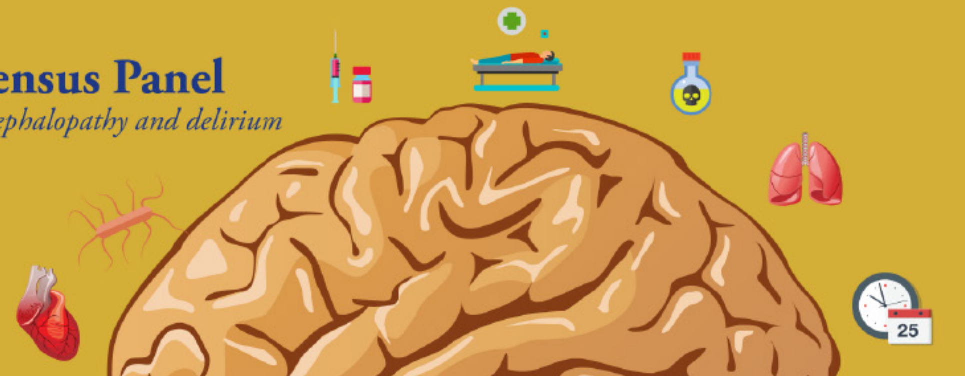

About the Panel

The Panel was founded by Arjen Slooter, Carsten Hermes and Thomas Ottens. As clinicians and researchers, we aim to address the question: "what to do when we've detected delirium?"

The Panel currently consists of 38 recognized field experts from Europe, Australia, Canada and the United States.

The main objective of the Panel is to come up with useful clinical decision algorithms, to support healthcare workers with a structural approach to detecting and managing underlying causes of acute encephalopathy and delirium

Assignment 1: Responses

Thank you very much for completing Assignment 1. We appreciate your time and effort. In this assignment, we asked you to respond to 4 general statements. They serve as the preamble to this project and were intended as a thermometer for the general mindset of the Panel.

Statement 1:
"Acute encephalopathy / delirium detection should be part of routine hospital care, especially in the elderly and patients with a vulnerability due to cerebral or cardiovascular disease, critical illness or substance abuse"

32 out of 35 responders fully agreed, 2 partially agreed and 1 partially disagreed

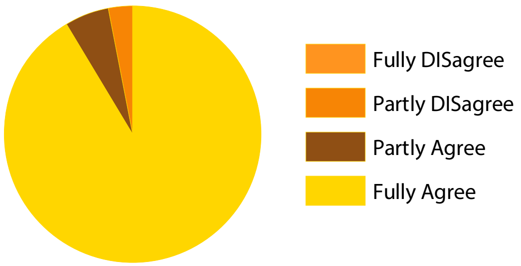

Statement 2:
"Early detection and monitoring of acute encephalopathy / delirium allows earlier detection and treatment of underlying diseases and disorders"

33 out of 35 responders fully agreed, 1 partially agreed and 1 partially disagreed

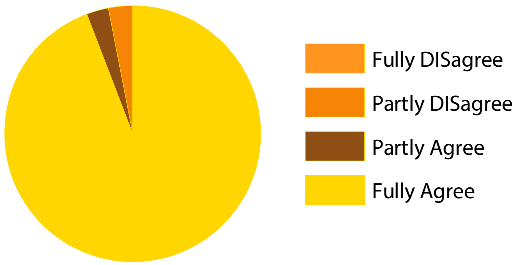

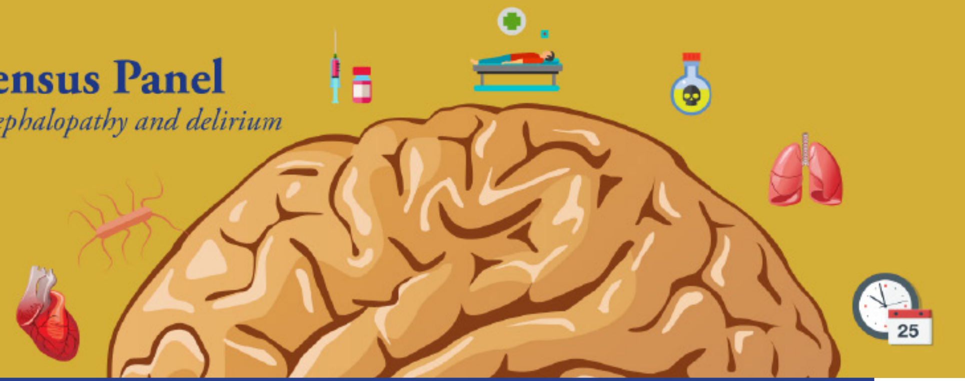

Assignment 1: Responses

Statement 3:
“Early detection of underlying diseases and disorders of acute encephalopathy/delirium may prevent unnecessary hospital morbidity and mortality”

26 of 35 responders fully agreed, 7 partly agreed and 2 partly disagreed.

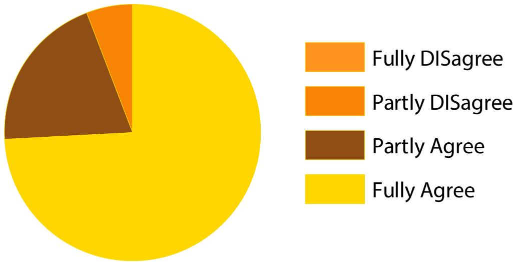

Statement 4:
“Early detection of acute encephalopathy / delirium allows earlier elimination of underlying causes. In turn, this may reduce encephalopathy / delirium burden, prevent long-term cognitive damage and reduce patient suffering”

26 of 35 responders fully agreed, 8 participants partly agreed and 1 partially disagreed

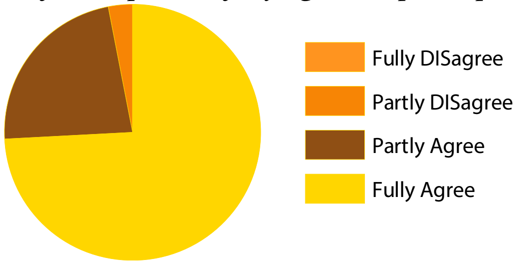

Assignment 1: Summary and Conclusions

The participation rate for this assignment was high (35/38), indicating that the panel acknowledges the importance of the topic.

There was a high level of expert consensus on each of the four preamble statements. The free text comments we received mostly concerned semantic adjustment suggestions, e.g. on the use of the term “substance abuse” and about the order in which delirium and acute encephalopathy should be mentioned.

The high level of agreement on the the pre-amble statements is promising for the result of future voting sessions on the delirium mangement algorithms.

Thank you very much for your participation in the first voting assignment. We will keep you updated!

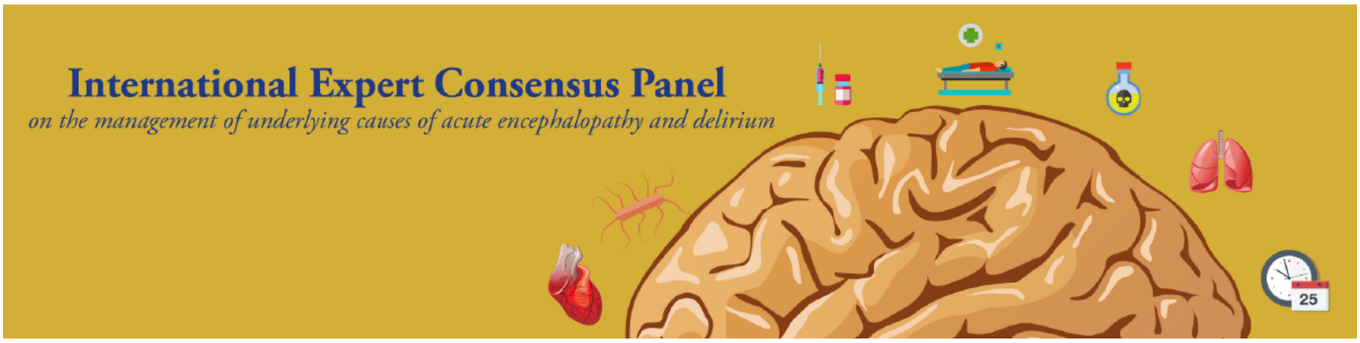

Report Assignment 2

Dear Panel Members,

Thank you for your continued support to the **International Expert Consensus Panel** on the management of underlying causes of acute encephalopathy and delirium.

We have processed your responses to the Ward Algorithm. We are happy to report that there was good agreement on many parts of the Algorithm. Within the Board, we have evaluated all of your feedback and reflected on how we could improve the content of the Ward Algorithm with it.

Despite the high level of agreement, we've decided to make some significant changes to improve the Ward Algorithm. We want to improve the Ward Algorithm as much as we can, because we consider it to be the blueprint for the other two Algorithms we plan to create at a later stage (ICU patients, cardiac surgery patients). Once all Algorithms are created, they may be presented together in printed and digital form, with accompanying explaining text. We intend to keep them alive and update them regularly. For more information, consult the project Charter.

In this report, we'll take you through the most important changes to the Ward Algorithm. You can find a **link to the updated Algorithm as well as the previous version** of the Ward Algorithm in the Online Voting Environment.

After reading the report, we ask you to **complete a Voting Assignment** on the changes we made. You may click here to access it: https://forms.gle/zjgprT4z2qP3TqBZ6

We thank you for your continued support to this project!

Sincerely,

Thomas Ottens
Carsten Hermes
Arjen Slooter

Part 1 : Algorithm Blocks

STEP 1

We have rephrased the wording of the first blocks in the Algorithm.

- Some responses we received seemed to indicate that the target population for this Algorithm wasn't clear enough. We have rephrased the intro block to make clear that this Algorithm is meant to be used for patients who are begin treated in regular hospital wards (and not, for example, a new patient presenting to the Emergency Department with a new condition), and whose delirium monitoring results indicate possible AE/delirium.
- There was **high level of agreement (89%)** on the statement on non-pharmacologic measures. This statement remains unchanged.
- We received valuable suggestions to improve the order of presentation of the different non-pharmacologic measures, and have updated the Card accordingly. (*See section on Reference Cards)*
- There was **high level of agreement (89%)** on the statement mentioned in STEP 1.
- We received valuable suggestions about the wording. We have rephrased the explaining text in this block to reflect the seriousness of some of the conditions that are in the differential diagnosis of acute changes in attention, arousal and cognition.
- We also received many suggestions that we used to improve the content of Reference Card B (*see, section on Reference Cards)*

Algorithm for patients in HOSPITAL WARDS (not in a monitored environment)

This algorithm is intended for patients whose monitoring results indicate possible acute encephalopathy / delirium during admission to a hospital ward. All patients should receive preventive non-pharmacologic measures, regardless of their cognitive state (**see Reference Card A**).

STEP 1: VERIFY your diagnosis - consider alternative diagnoses
Perform a careful **physical examination** for signs of other acute, potentially life-threatening neurologic disorders that may appear similar to acute encephalopathy / delirium. See **Reference Card B** for guidance.

CONFIDENTIAL

STEP 2:

Although there was a **reasonable level of agreement (66%)** on the content of STEP 2, we received many comments. Some Panel members requested more specific diagnoses or laboratory tests be added, others considered the lists too long.

After thorough reflection and evaluation by the Board members, we have decided to simplify and restructure the recommendations in STEP 2, rather than adding more and more diagnoses, signs, symptoms and tests. The general idea here is, that the Algorithm should remain concise and shouldn’t take the role of a medical textbook.

This may also avoid unwanted effects of using the Algorithm, such as unnecessary / irrelevant diagnostic tests.

Finally, we harmonized the structure of the recommendations.

The recommendations are now grouped as “infection”, "electrolytes/metabolic disturbances", "PADIS" and “drugs”.

STEP 2: Identify and treat COMMON underlying causes
Vulnerable patients (e.g. the elderly, patients with neurodegenerative disorders) may develop delirium even from mild disturbances.

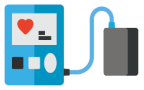

Evaluate vital signs
Screen for circulatory and respiratory insufficiency. Use an Early Warning Score system to monitor changes in vital signs over time.

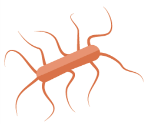

Evaluate and treat for infection / sepsis
Screen for common infections, consider blood cultures. Sepsis may be afebrile in elderly and immunocompromised patients.

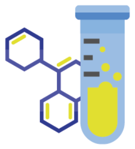

Evaluate and correct metabolic disorders
Dehydration, electrolyte imbalance (sodium, magnesium, calcium), glucose level, metabolic acidosis, kidney or liver dysfunction.

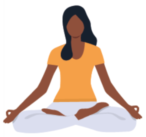

Evaluate and treat pain, anxiety, discomfort, immobility and sleep disturbances
Consider occult pain, bladder retention and obstipation (see **Reference Card C**).
Monitor pain with behavioral scales (e.g. Behavioral Pain Scale, BPS) in non-verbal patients.

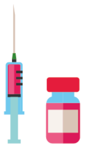

Review drugs and intoxications
- Evaluate all medication and possible interactions.
- Consider intoxication with or withdrawal from nicotine, alcohol and recreational drugs.
- Review sedatives and opioids: these may trigger and prolong delirium.
- Anticholinergic drugs: these may trigger and prolong delirium (see **Reference Card D**).
- Consider checking blood levels of medication.

CONFIDENTIAL

STEP 3:

There was a **relatively low level of agreement (46%)** on the content of STEP 3, concerning symptomatic treatment.

Most of the comments on this section concerned practice variations across the different parts of the world where Panel members practice. After discussion in the Board, we removed specific drug names and only refer to drug classes. Again, we aimed for a concise, practical Algorithm. By simplifying, we try to avoid the “textbook effect”.

STEP 3: Symptomatic treatment (in a non-monitored environment)

Symptomatic treatment should be individualized, focusing on predominant symptoms. Only apply physical restraints if strictly necessary and if this can be done safely.

- **Psychomotor agitation and anxiety**: consider antipsychotics if agitation hinders nursing or poses a safety risk. Reserve benzodiazepines as a rescue treatment for severe agitation or anxiety, as benzodiazepines may maintain delirium. Low dose benzodiazepines may be indicated in patients with alcohol withdrawal.

- **Hallucinations, delusions:** consider antipsychotics if these cause discomfort, anxiety or agitation.

- **Somnolence, apathy, psychomotor slowing:** reduce sedatives, start mobilization, physical therapy, create a stimulating environment (music, family visits).

CONFIDENTIAL

STEP 4:

There was a **reasonable level of agreement (57%)** on the content of STEP 4: monitor effect. We noticed that the text caused confusion and, to some, suggested we advocate a full EEG as a means of monitoring. The original intention was to suggest using either a clinical delirium tool like CAM, or an automated 1-channel EEG device - not a full EEG.

To avoid confusion, we decided to simplify the text to “monitor symptoms and treatment effect”.

There was a **high level of agreement (71%)** on the content of the boxes after STEP 4. However, we received some suggestions that we found very useful. After discussion in the Board, we decided to use the suggestions to update this section.

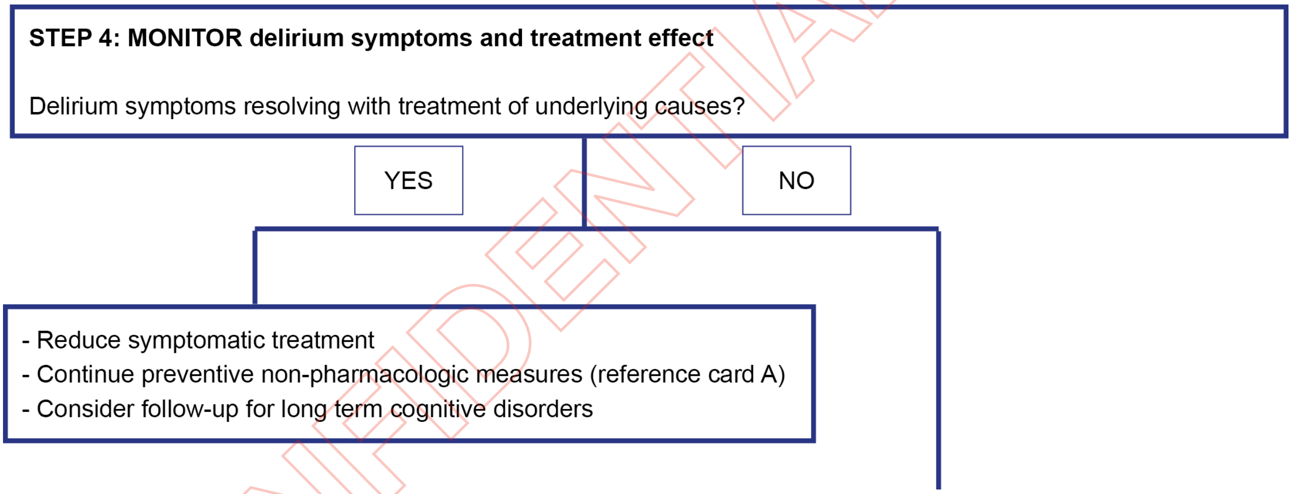

CONFIDENTIAL

STEP 5:

There was a **high level of agreement (77%)** on the content of "STEP 5: search for less common underlying causes”.

Still, we received a very high number of free text comments to this section. Again, many Panel members suggested to remove certain items because they considered them not relevant enough to be presented, and others suggested adding items they considered missing.

After discussion between the Board members, we propose to re-structure this section of the Algorithm.

Originally, STEP 5 contained 4 blocks with suggestions, and then a separate block that referred to a Reference Card with even rarer possible underlying causes. (This section had a **high level of agreement (86%)**)

After reading all the Panel’s comments, the original division seemed too arbitrary. To keep the Algorithm concise, we moved all less common underlying causes to one Reference Card “E”. (See section on Reference Cards).

STEP 5: search for LESS COMMON underlying causes
Delirium may persist despite optimal treatment of the underlying cause. If none of the common underlying causes is present, or delirium persists or worsens under treatment, consider less common underlying causes. These are shown in **Reference Card E**.

CONFIDENTIAL

Reference Cards

All the variants of the Algorithms (ward, ICU, cardiac surgery) refer to a set of Reference Cards, containing relevant information that is too extensive to be put into the Algorithm itself. When the Algorithms are followed step-by-step, the user is prompted to look at the Reference Cards in alphabetic order.

Because of the changes we made to the Algorithm, the order of the Reference Cards had to be changed accordingly.

Overview, in alphabetic order:

- **Reference Card A: non-pharmacologic preventive measures**
- **Reference Card B: alternative diagnoses in patients with possible acute encephalopathy / delirium**
- **Reference Card C: Occult sources of pain and discomfort in non-communicating patients**
- **Reference Card D: Drugs with strong anticholinergic effects**
- **Reference Card E: Less common underlying causes of acute encephalopathy / delirium**

Reference Card A: non-pharmacologic preventive measures
This card card has suggestions for 1) reassurance and reorientation 2) early mobilization and physical therapy 3) cognitive stimulation during the day 4) minimal disturbance during the night 5) and some general preventive measures and remarks .

The card has been updated using suggestions from the Panel.

Reference Card A: Non-pharmacologic preventive measures

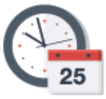

Verbally reassure the patient and support reorientation. Provide a well-visible clock and calendar. Ensure the patient has access to their glasses and hearing aids. Provide structure with a daily schedule of activity and resting hours.

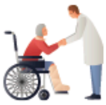

Start mobilization and physical therapy as early as possible. Provide a schedule tailored to the indiviual patients' needs.

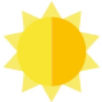

Create a stimulating environment during daytime. Expose patients to bright light during the day. Encourage listening to music and other stimulating activities. Encourage contact with loved ones. Online social contacts may be helpful to certain patients.

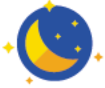

Define night-time clearly. During night-time, avoid medical and nursing care activities that are not strictly necessary. If desired by the patient, provide a sleep mask and ear plugs. Limit noise and light exposure to an absolute minimum during night-time.

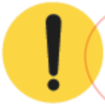

Ensure adequate hydration, nutrition and bowel care
Avoid prolonged fasting for procedures.
Keep the use of physical restraints to an absolute minimum.
Avoid routine pre-medication for anesthesia.

CONFIDENTIAL

Reference Card B: Alternative diagnoses in patients with possible acute encephalopathy / delirium

This card is meant to help the user distinguish between AE/delirium and other causes that require immediate attention. Distinguishing between AE/delirium and these disorders (stoke, meningitis, seizures, Wernicke, serotonin syndrome and malignant neuroleptic syndrome) has direct consequences for further diagnostic work-up and treatment.

The card has been updated using suggestions from the Panel

Reference Card B: Alternative diagnoses in patients with possible acute encephalopathy / delirium

Perform a **physical examination** and consider the differential diagnosis.

- Focal neurological deficits: broad differential diagnosis, including **stroke and other structural brain pathology**
Consult a neurologist, brain imaging

- Fever (or immune deficiency) and/or meningeal irritation: **meningitis / encephalitis**
Consult a neurologist, brain imaging, lumbar puncture

- History of seizures/epilepsy or other brain disorders, abnormal eye movements, automatisms or muscle twitching: (non-convulsive) **seizures**
Consult a neurologist, EEG

- Nystagmus, ocular palsy, history of undernourishment or alcohol abuse: **Wernicke encephalopathy**
Treat with high dose thiamine and other vitamins

- Autonomic hyperactivity (shivering, hyperthermia, mydriasis, hypertension) with neuromuscular features (tremor, myoclonus, hyperreflexia): **serotinin syndrome**
Discontinue serotonergic drugs, consider anti-serotonergic therapy

- Hyperthermia, autonomic instability, rigidity, tremor, increased creatine kinase: **malignant neuroleptic syndrome** (especially after starting neuroleptic drugs)
Discontinue neuroleptic drugs, consider dopamine agonist, symptomatic treatment, monitor creatine kinase

CONFIDENTIAL

Reference Card C: Occult sources of pain and discomfort in non-communicating patients
This card is intended to help caregivers look for causes of discomfort. This should be especially helpful in patients on mechanical ventilation, patients receiving sedation, and those unable to communicate - also in normal ward patients.

Reference Card C has not been shown in any of the voting assignments yet.

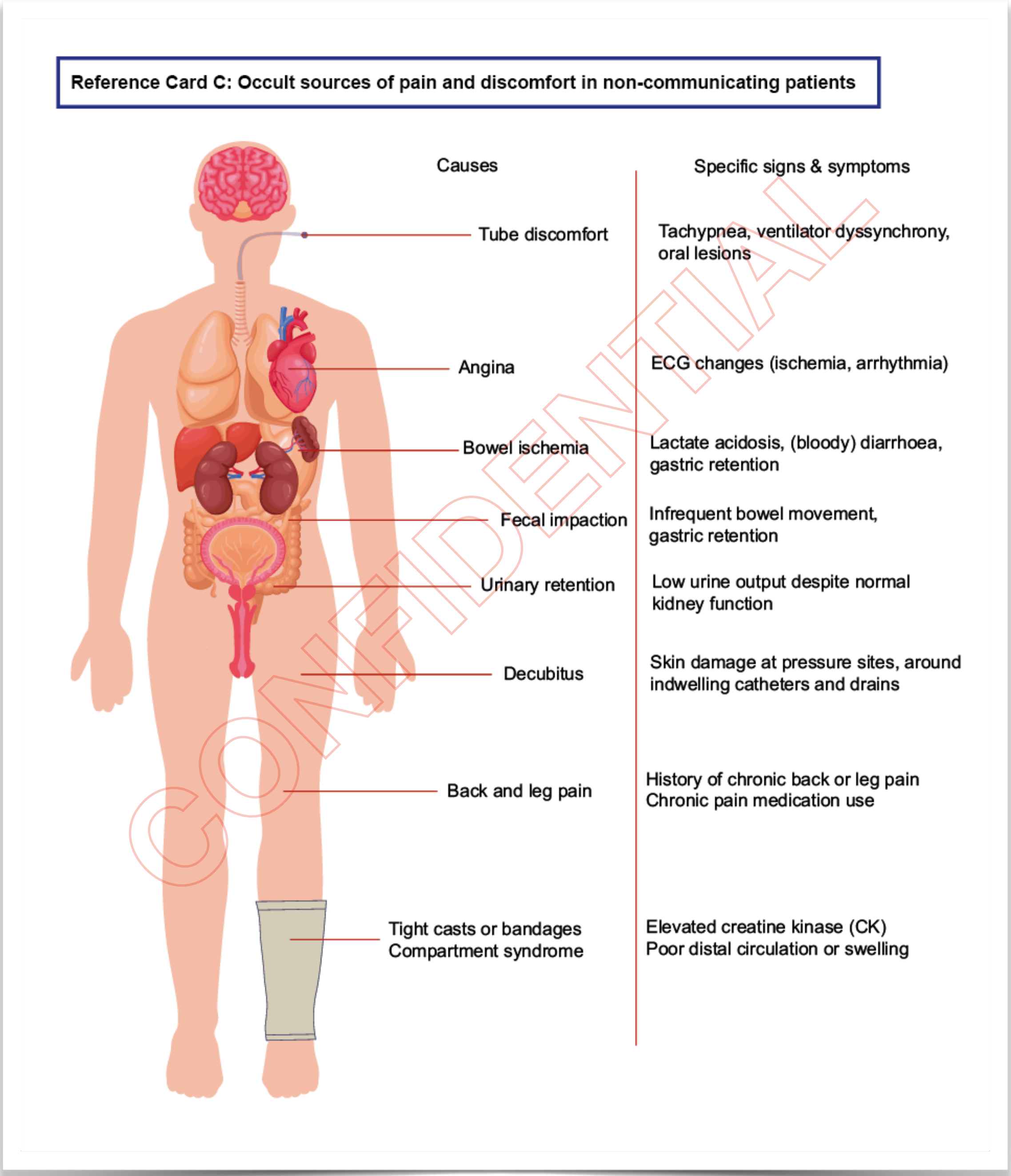

Reference Card D: Drugs with strong anticholinergic effects
This card helps the caregiver in evaluating the patients medication. It is based on the Anticholinergic Drug Scale.

Reference Card D has not been shown in any of the voting assignments yet.

Reference Card D: Drugs with strong anticholinergic effects

The Anticholinergic Drug Scale (ADS) is an expert classification of anticholinergic drug effects. This is a list of commonly used drugs with anticholinergic effects. **This is not a complete list.** Less potent anticholinergic drugs may still cause relevant adverse effects, for example when combined. Especially in patients with polypharmacy, the use of online interaction tools can be useful.

Anticholinergic adverse effects include dry mouth and eyes, constipation, tachycardia, urine retention and several neurocognitive effects (forgetfullness, agitation, paranoia and delirium). Risk factors for developing anticholinergic adverse effects are advanced age and dementia.

CONFIDENTIAL

Level 3
Anticholinergic effects very likely

Amitriptyline
Atropine
Clemastine
Clomipramine
Chlorpromazine
Clemastine
Clomipramine
Clozapine
Darifenacin
Desipramin
Diphenhydramine
Hydroxyzine
Hyoscyamine
Imipramine
Meclozine
Nortriptyline
Oxybutinin
Promethazine
Scopolamine
Tolterodine
Trimipramine

Level 2
Anticholinergic effects likely

Carbamazepine
Cimetidine
Cyproheptadine
Disopyramide
Meperidine
Oxcarbazepine
Pimozide
Ranitidine

Reference Card E: Less common underlying causes of acute encephalopathy / delirium

Based on the comments of the Panel, we decided to remove the - somewhat arbitrary - distinction between the less common underlying causes mentioned in STEP 5 of the original Algorithm, and the “very uncommon causes” originally shown in a Reference Card.

This resulted in Reference Card E. This way, the Algorithm remains concise.

With feedback from the Panel, some of the endocrine/metabolic disorders and central nervous system disorders were changed, and a section on psychiatric disorders was added.

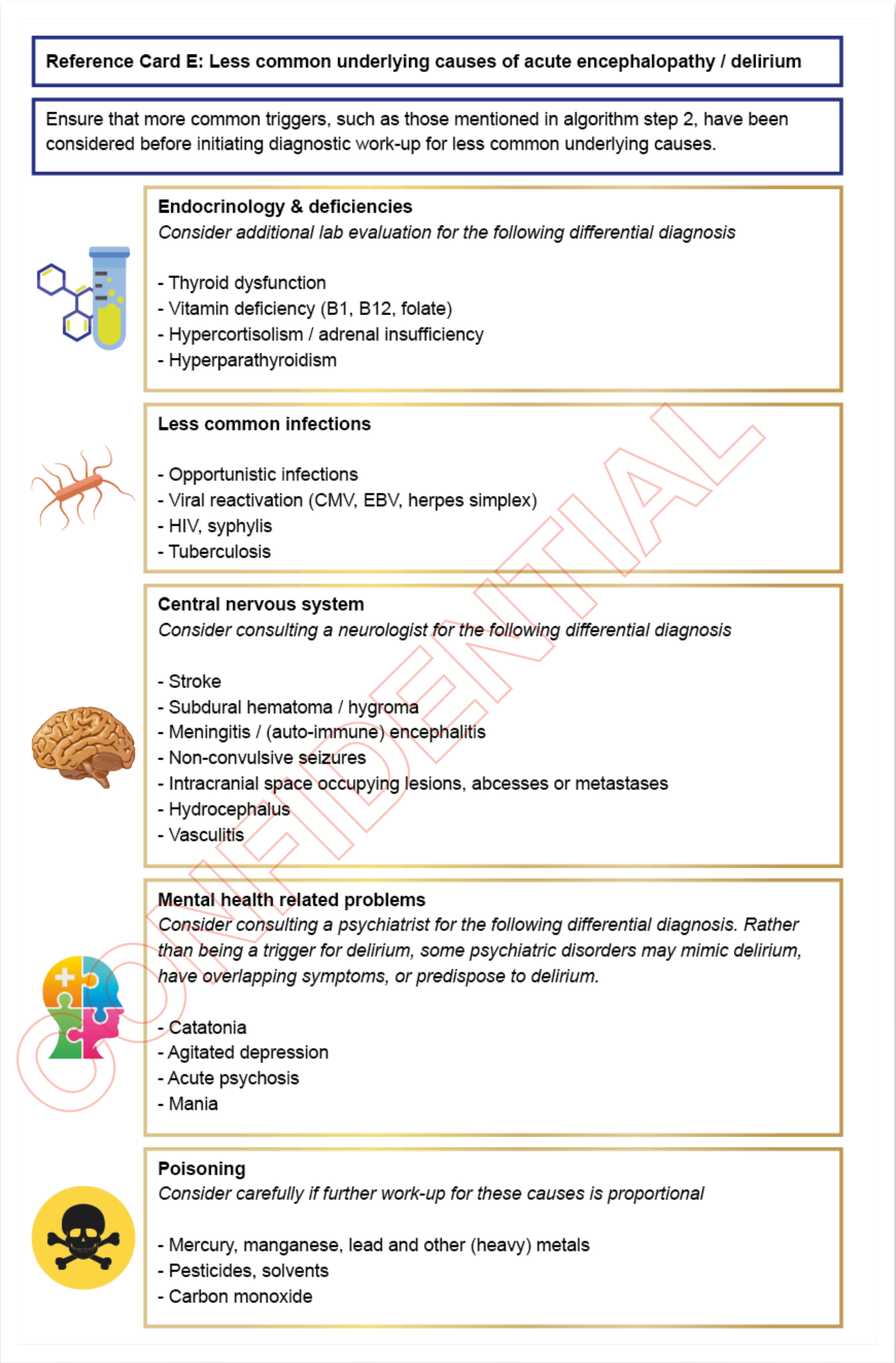

Reference Card E: Less common underlying causes of acute encephalopathy / delirium

Ensure that more common triggers, such as those mentioned in algorithm step 2, have been considered before initiating diagnostic work-up for less common underlying causes.

Endocrinology & deficiencies
Consider additional lab evaluation for the following differential diagnosis

- Thyroid dysfunction
- Vitamin deficiency (B1, B12, folate)
- Hypercortisolism / adrenal insufficiency
- Hyperparathyroidism

Less common infections

- Opportunistic infections
- Viral reactivation (CMV, EBV, herpes simplex)
- HIV, syphylis
- Tuberculosis

Central nervous system
Consider consulting a neurologist for the following differential diagnosis

- Stroke
- Subdural hematoma / hygroma
- Meningitis / (auto-immune) encephalitis
- Non-convulsive seizures
- Intracranial space occupying lesions, abcesses or metastases
- Hydrocephalus
- Vasculitis

Mental health related problems
Consider consulting a psychiatrist for the following differential diagnosis. Rather than being a trigger for delirium, some psychiatric disorders may mimic delirium, have overlapping symptoms, or predispose to delirium.

- Catatonia
- Agitated depression
- Acute psychosis
- Mania

Poisoning
Consider carefully if further work-up for these causes is proportional

- Mercury, manganese, lead and other (heavy) metals
- Pesticides, solvents
- Carbon monoxide

CONFIDENTIAL

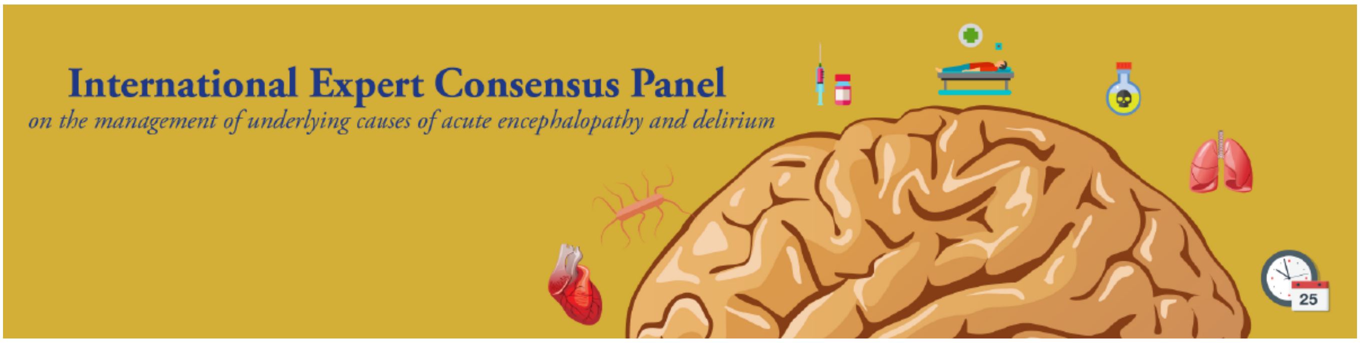

Report Assignment 3

Dear Panel Members,

Thank you for your continued support to the **International Expert Consensus Panel** on the management of underlying causes of acute encephalopathy and delirium.

In Assignment 3, you were asked to react to an updated version of our “Ward Algorithm”. Together with all the Reference Cards, this algorithm forms the basis of our project.

We are happy to report that the majority of our 38 panel members agreed with the proposed updates. Once again, we received some valuable suggestions. We made changes to some of the wording in the Algorithm, and to the look of the algorithm in some places.

We consider the Ward Algorithm to be in the final concept stage for now. In this report, we’ll briefly inform you on the results of Assignment 3. You can access the concept versions online via this link. Soon, we’ll continue with the **Algorithm for ICU patients.** You will receive an invitation in a separate e-mail.

We thank you for your continued support to this project!

Sincerely,

Thomas Ottens
Carsten Hermes
Arjen Slooter

Response rate:

32 of the 38 panel members have completed Assignment 3. We received several requests to extend the deadline. Non-responders have received two e-mail reminders.

Part 1 : Algorithm Blocks

The Ward Algorithm was adapted to the Panel comments where necessary. The concept version is available online (click here). The concept version is confidential, please do not share or use the algorithm before it is officially released.

Starter Block and STEP 1

There was a **high level of agreement (88%)** with the updates to the starter block and STEP 1. **88% of the responders agreed** with the specific updates to STEP 1 (VERIFY your diagnosis, consider alternative diagnoses).

No further changes have been made to this section. The level of agreement on this section was the same as in Assignment 2.

STEP 2

There was a **high level of agreement (72%)** with the updates to STEP 2: Identify and treat COMMON underlying causes.

We changed the pictogram for “infection” because a panel member specifically requested this

No further changes have been made to this section. The level of agreement on this section was higher than in Assignment 2 (66%).

STEP 3

There was a **reasonable level of agreement (66%)** with the updates to STEP 3: Symptomatic treatment.

Most of the comments concerned the choice of words where treatment with benzodiazepines are mentioned. We made minor changes to the wording in this section.

The level of agreement was higher than in Assignment 2, when it scored a relatively low level of agreement (46%). After discussion in the Board, we consider this section to have enough agreement (> 65% threshold) at this time and consider it as a definitive concept.

STEP 4

There was a **high level of agreement (84%)** with the updates to STEP 4: MONITOR delirium symptoms and treatment effect.

No further changes have been made to this section. The level of agreement on this section was higher than in Assignment 2 (57%).

STEP 5

There was a **high level of agreement (84%)** with the updates to STEP 5: search for LESS COMMON underlying causes.

In Assignment 2, we received many free text comments despite a relatively high level of agreement (77%). The section was therefore drastically changed. Apparently, this resonated well

with the respondents, considering the high level of agreement with the proposed updates. No further changes have been made to this section.

Reference Card A: Non-pharmacologic preventive measures

There was a **high level of agreement (88%)** with the updates to this card. No further changes have been made to this card.

Reference Card B: Alternative diagnoses in patients with possible acute encephalopathy / delirium

There was a **high level of agreement (72%)** with the updates to this card. No further changes have been made to this card.

Reference Card C: Occult sources of pain and discomfort in non-communicating patients

This card was presented to the panel for the first time. There was a **high level of agreement (78%)** with the content of this card.

Some minor changes were made to the card based on the respondent's suggestions.

Reference Card D: Drugs with strong anticholinergic effects

This card was presented to the panel for the first time. There was a **high level of agreement (75%)** with the content of this card.

Some text errors on the card have been corrected. Because of the international variation in drug availability and relevance, the Board is considering an alternative to this card. Once this has been designed, it will be presented to the Panel in a future voting assignment. The card is considered definitive concept for now.

Reference Card E: Less common underlying causes of acute encephalopathy / delirium

This was a new card that was created together with the update to Algorithm STEP 5. There was a **high level of agreement (75%)** with the content of this card.

Based on respondent's suggestions, we added dementia to the list of central nervous system causes and changed the wording of the block with Mental Health Related Problems.

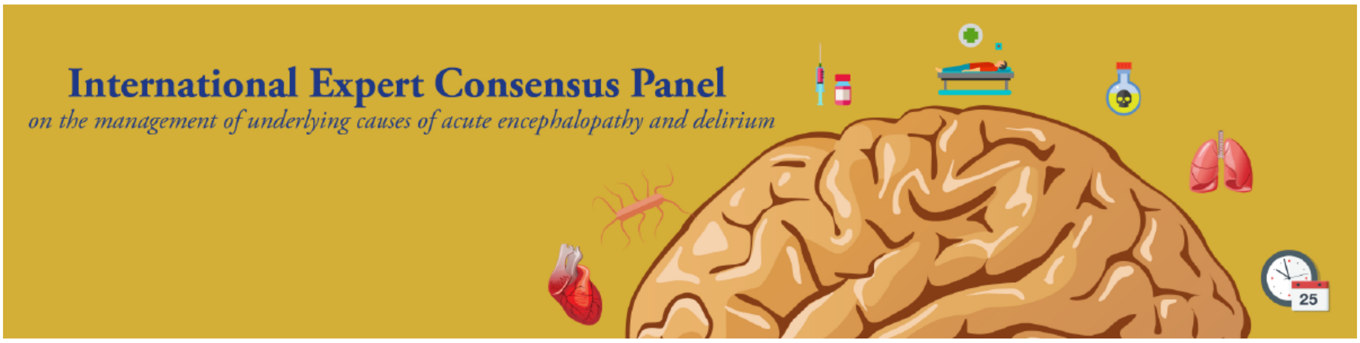

Report Assignment 4

Dear Panel Members,

Thank you very much for your continued support to the **International Expert Consensus Panel** on the management of underlying causes of acute encephalopathy and delirium.

In Assignment 4, you were asked to react to the algorithms for **Cardiac Surgery and ICU patients**.

Once again, the level of agreement with the algorithm content was excellent. As always, we received some valuable suggestions. This time, we felt it was only necessary to change the wording in the Algorithms. These changes are summed up in the report for your reference.

The next step of this project is the production phase. This step consists of:

1. Thorough language checks by native speakers
2. Preparation of a manuscript for publication
3. Production of materials such as a website from where the medical community can access the algorithms

In one final online Assignment, we will ask your formal **endorsement** for the complete set of algorithms, including a brief introduction, disclaimers, and all reference cards.

We thank you for your continued support to this project, and look forward to the final step!

Sincerely,

Thomas Ottens
Carsten Hermes
Arjen Slooter

Response rate:

Despite several reminders, this time only 29 of the 38 panel members (76%) responded to Assignment 4. One of the 29 respondents indicated they did not wish to comment on the Cardiac Surgery or ICU algorithms. Thus, 28 responses were analysed (73.7%).

Cardiac Surgery (CS) Algorithm

Starter Block and STEP 1

We did not include a question on STEP 1, because it is identical across the different algorithms.

STEP 2

There was a **100% agreement** with way we designed STEP 2: Identify and treat COMMON underlying causes for the CS algorithm.

However, some Panel members commented on the order of the blocks in STEP 2 in the ICU algorithm, we have decided to rearrange the blocks in both the CS and ICU algorithm, so they are more in a typical **ABCD+ type order**.

The order for the CS and ICU algorithms are now:
A & B: evaluate airway and respiratory tract)
C: evaluate circulatory tract)
D: evaluate PADIS
Followed by the blocks for infection, metabolic disorders and drugs/intoxications/withdrawal

In the introductory text that will accompany the algorithms, we will stress that users are encourages to evaluate patients with AE/delirium for all blocks, not necessarily in this order.

Some Panel members disliked the icon for the PADIS-block; we changed it to a different, gender neutral symbol (the comedy/tragedy masks symbolise mood, anxiety levels, pain/suffering etc).

The final version of STEP 2 in the CS algorithm now looks like this:

Updated STEP 2 of the Cardiac Surgery algorithm

STEP 2: Identify and treat COMMON underlying causes
Vulnerable patients (e.g. the elderly, patients with neurodegenerative disorders) may develop delirium even from mild disturbances.

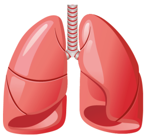

Airway & respiratory tract
Assess for airway obstruction, increased work of breathing, pain worsening with breathing, asymmetric chest excursions. Consider pneumothorax, pulmonary edema, pneumonia and pulmonary embolism.

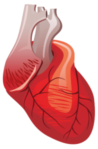

Circulation
Assess circulation by physical examination and vital signs.
Consider low cardiac output (due to arrhythmia, hypovolemia, bleeding, heart failure, tamponade)
Consider myocardial ischemia. Check ECG, Hb, lactate, cardiac ischemia markers.

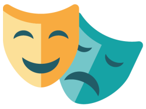

Pain, anxiety, discomfort, immobility and sleep disturbances
Consider occult pain, bladder retention and constipation (see **Reference Card C**).
Monitor pain with behavioral scales (e.g. Behavioral Pain Scale, BPS) in non-verbal patients.

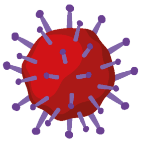

Infection / sepsis
Inspect all wounds and indwelling lines/drains. Consider infections of prosthetics and endocarditis.
Measure CRP, leukocytes and/or procalcitonin, consider blood- and other relevant cultures.

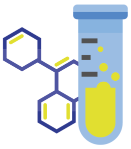

Metabolic disorders
Assess for dehydration, electrolyte imbalance (sodium, magnesium, calcium), glucose level, metabolic acidosis, kidney or liver dysfunction.

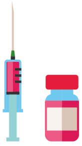

Drugs and intoxications
- Evaluate all medication and possible interactions.
- Consider intoxication with or withdrawal from nicotine, alcohol and recreational drugs.
- Review sedatives and opioids: these may trigger and prolong delirium.
- Anticholinergic drugs: these may trigger and prolong delirium (see **Reference Card D**).
- Consider checking blood levels of medication.

Updated order to ABCD-style, changed symbol for PADIS

CONFIDENTIAL // NOT FOR PUBLICATION

STEP 3

There was a **reasonable level of agreement (66%)** with the content in STEP 3: Symptomatic treatment.

We received various suggestions to improve the text in this step. Some of the comments suggested changes that would involve significant increases in the number of words. As a board, we discussed how to best process these comments. In line with the way we have handled such comments in previous Assignments, we have decided to keep the algorithms texts brief, rather than lengthy and detailed. In the same way, we handled comments that suggested changes that would make the algorithm text repeat itself. After all, the algorithms are intended as a cognitive aid, and are not intended to replace adequate medical/nursing training or be used as a textbook-like resource.

After reading and summarising the comments in this section, we distilled the general idea that some Panel members think that readers should be more firmly reminded that

- Drug treatments should only be initiated if non-pharmacologic measures provide insufficient symptom control
- There are no generally accepted drug treatments for hypoactive symptoms, and these should be treated exclusively non-pharmacologically

Because non-pharmacologic measures are all summed up in Ref Card A, we have decided to refer to Ref Card A in the text preceding suggested drug treatments.

Finally, some panel members pointed out that clonidine is not as widely used as dexmedetomidine. We therefore changed the order in the text to "dexmedetomidine or clonidine".

The updated version of STEP 3 for the CS algorithm now looks like this:

Updated STEP 3 of the CS algorithm

STEP 3: Symptomatic treatment
Symptomatic treatment should be individualized, focusing on predominant symptoms. Only initate drug treatments for hyperactive and psychotic symptoms when non-pharmacologic measures (see **Reference card A**) provide insufficient relief. The choice of symptomatic drug treatments depends on the environment of the patient.

- Psychomotor agitation and anxiety: consider **antipsychotics** if agitation hinders nursing or poses a safety risk. *In monitored environments (ICU, HDU, PACU), consider* ***dexmedetomidine*** *or* ***clonidine***. Reserve benzodiazepines as a rescue treatment for severe agitation or anxiety, as benzodiazepines may contribute to ongoing delirium. Benzodiazepines may be indicated in patients with alcohol withdrawal.

- Hallucinations, delusions: consider **antipsychotics** if these cause discomfort, anxiety or agitation.

- Somnolence, apathy, psychomotor slowing: reduce sedatives, start mobilization, physical therapy, create a stimulating environment (family visits, music, therapeutic activities).

CONFIDENTIAL // NOT FOR PUBLICATION

STEP 4 and 5

We only invited free-text comments to STEP 4: Monitor delirium symptoms and treatment effect and STEP 5: search for LESS COMMON underlying causes, because these blocks are identical across the algorithms.

Using these comments, we improved the wording in this section ***across all algorithms*** as follows

- we changed the title of STEP 4: to "MONITOR the cognitive state and effect of initiated treatments"
- We added "Assess frequently, using validated scales (RASS, NRS, CAM, etc) according to local guidelines.

In the block after "yes"

- to "reduce symptomatic drug treatments" we added "to the lowest effective dose"
- We added "re-assess underlying causes / triggers daily"

The updated version of this section now looks like this, across all algorithms:

Updated STEP 4 and 5 (for all algorithms)

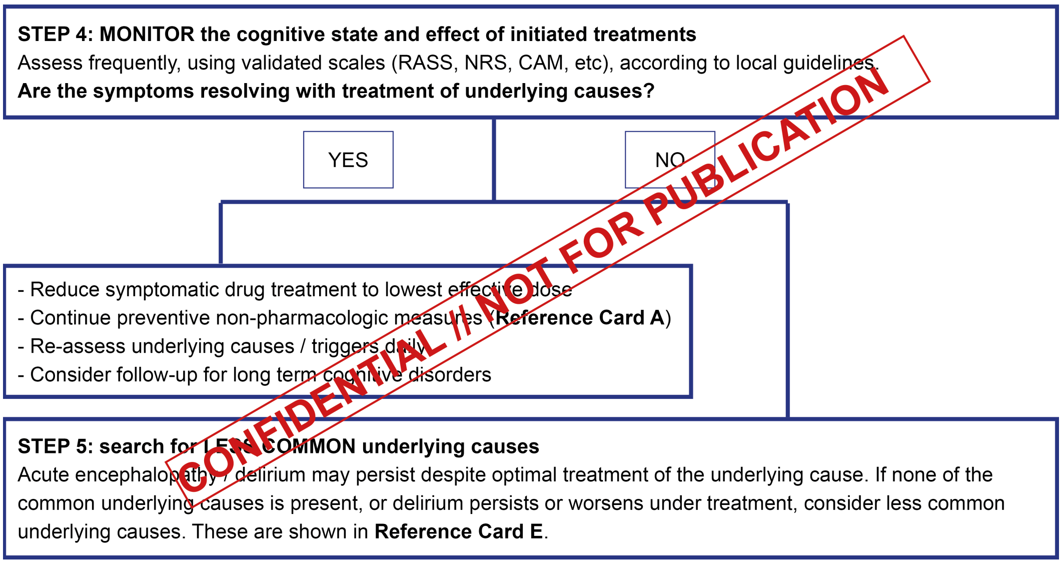

CONFIDENTIAL // NOT FOR PUBLICATION

Updated title and text for STEP 4, added suggestions to "YES" block

ICU Algorithm

Starter Block and STEP 1

We did not include a question on STEP 1, because it is identical across the different algorithms.

STEP 2

There was a **high level of agreement (82%)** with way we designed STEP 2: Identify and treat COMMON underlying causes for the CS algorithm. As mentioned above, we only changed the order of the blocks to the ABCD+ style and changed the icon for PADIS.

The final version of STEP 2 in the ICU algorithm now looks like this:

Updated STEP 2 of the ICU algorithm

STEP 2: Identify and treat COMMON underlying causes
Vulnerable patients (e.g. the elderly, patients with neurodegenerative disorders) may develop delirium even from mild disturbances.

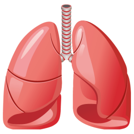

Airway & respiratory tract
Assess the respiratory tract by physical examination and monitoring parameters. Check arterial blood gas. Consider chest imaging and respiratory cultures.
Consider pneumothorax, pulmonary edema, pneumonia and pulmonary embolism.
In mechanically ventilated patients, assess and optimize ventilator settings. Consider high work of breathing, overexertion, tube obstruction, dyssynchrony, tube discomfort, sinusitis.

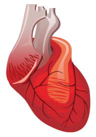

Circulatory tract
Assess circulatory system by physical examination and monitoring parameters.
Consider low cardiac output (due to arrhythmia, hypovolemia, bleeding, heart failure, tamponade)
Consider myocardial ischemia. Check ECG, Hb, lactate, cardiac ischemia markers.

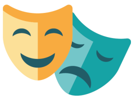

Pain, anxiety, discomfort, immobility and sleep disturbances
Consider occult pain, bladder retention and constipation (see **Reference Card C**).
Monitor pain with behavioral scales (e.g. Behavioral Pain Scale, BPS) in non-verbal patients.

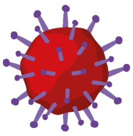

Infection / sepsis
Screen for common infections, consider blood cultures. Sepsis may be afebrile in ICU patients.
Consider infections of indwelling catheters and drains, implanted prosthetics and endocarditis.

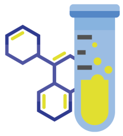

Metabolic disorders
Imbalance of sodium, ionized calcium or glucose, metabolic acidosis, kidney dysfunction (uremia) or liver dysfunction (elevated bilirubin, liver enzymes or ammonia)

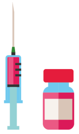

Drugs and intoxications
- Evaluate all medication and possible interactions.
- Consider intoxication with or withdrawal from nicotine, alcohol and recreational drugs.
- Review sedatives and opioids: these may trigger and prolong delirium.
- Anticholinergic drugs: these may trigger and prolong delirium (see **Reference Card D**).
- Consider checking blood levels of medication.

CONFIDENTIAL // NOT FOR PUBLICATION

Updated order to ABCD-style, changed symbol for PADIS

STEP 3

There was a **reasonable level of agreement (64%)** with the content in STEP 3: Symptomatic treatment.

We combined comments from both CS and ICU algorithms and improved this step as described above. The final version for the ICU algorithm looks like this:

Updated STEP 3 of the ICU algorithm

STEP 3: Symptomatic treatment
Symptomatic treatment should be individualized, focusing on predominant symptoms. Only initate drug treatments for hyperactive and psychotic symptoms, when non-pharmacologic measures (**Reference Card A**) provide insufficient relief. Only apply physical restraints if strictly necessary.

CONFIDENTIAL // NOT FOR PUBLICATION

- **Psychomotor agitation and anxiety**: start with **dexmedetomidine** or **clonidine**, titrated to effect. Consider adding **antipsychotics** if agitation hinders nursing or poses a safety risk. Reserve benzodiazepines as a rescue treatment for severe agitation or anxiety, as benzodiazepines may contribute to ongoing delirium. Benzodiazepines may be indicated in patients with alcohol withdrawal.

- **Hallucinations, delusions:** consider **antipsychotics** if these cause discomfort, anxiety or agitation.

- **Somnolence, apathy, psychomotor slowing:** reduce sedatives, start mobilization, physical therapy, create a stimulating environment (family visits, music, therapeutic activities).

STEP 4 and 5

Step 4 and 5 are identical across the algorithms.

Further remarks:

We received comments across all questions regarding involvement of family. Although it was already mentioned on Reference Card A, we decided to change the wording of the Reference Card so this suggestion becomes more prominent.
