# Supplementary material for: The Delphi Delirium Management Algorithms. A practical tool for clinicians, the result of a modified Delphi expert consensus approach
Source: Delirium (Bielef). Author manuscript; Available in PMC 2024 Feb 12. (PMC10861222; doi:10.56392/001c.90652)
Supplement: Additional File 2 [file NIHMS1959412-supplement-Additional_File_2.pdf]

The Delphi Algorithms for Management of Delirium and Acute Encephalopathy

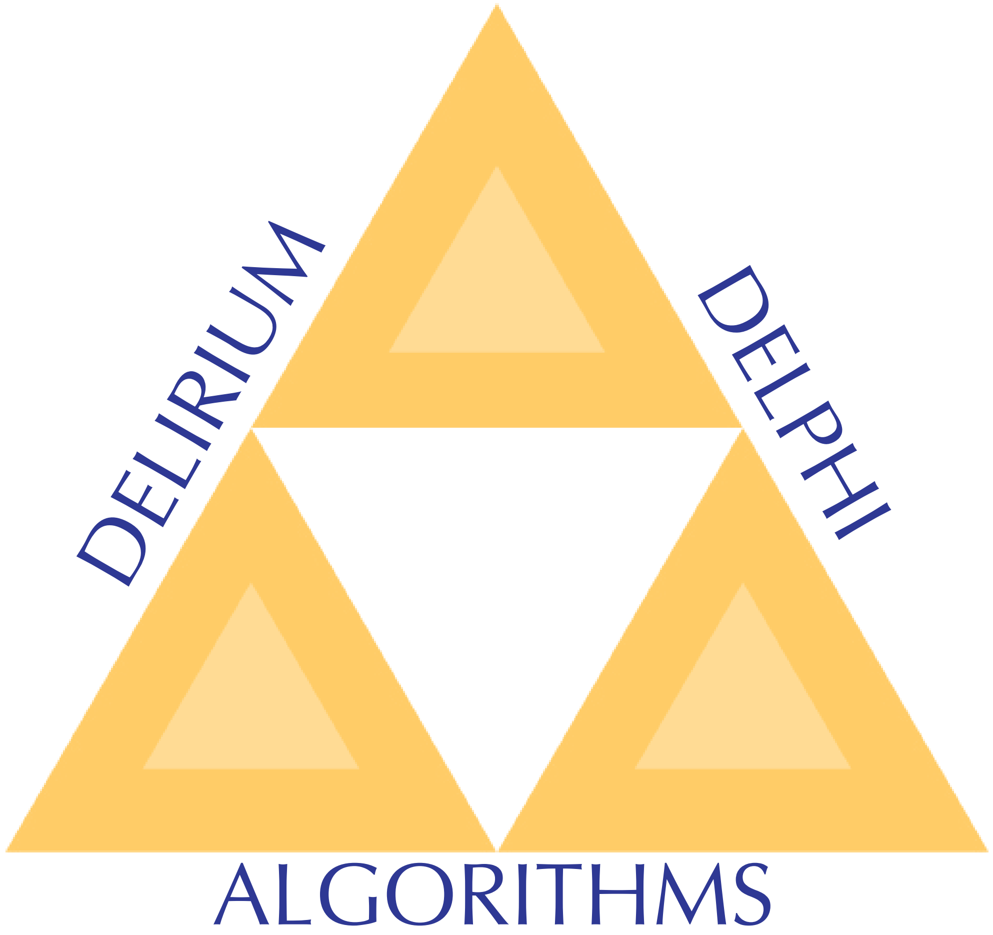

This booklet contains algorithms for the management of underlying causes of (subsyndromal) delirium as expression of acute encephalopathy in hospitalized adults/ The algorithms are intended to be used by licensed health care practitioners.

Contents:

Note to users 2
Patients in hospital wards 4
Patients after cardiac surgery 6
Patients in intensive care units 8
Reference card A: Nonpharmacologic preventive measures 10
Reference card B: Alternative diagnoses 11
Reference card C: Potential sources of pain in non-communicating patients 12
Reference card D: Less common underlying causes of delirium 13
Reference card E: Drugs with strong anticholinergic effects 14
List of abbreviations 15

Note to users

This set of clinical algorithms was developed by the I*nternational Expert Consensus Panel on the management of underlying causes of delirium*. This interdisciplinary group of international delirium experts intended to produce algorithms to assist healthcare providers (HCP) in taking care of adult patients with delirium or acute encephalopathy (AE). The algorithms can be used to structure the approach of the patient who develops delirium or AE. It provides suggestions on preventive measures, differential diagnosis, an approach to detection of underlying causes and how to prioritize these, treatment of symptoms, and follow-up.

There are algorithms for three categories of hospitalized adult patients:

Algorithm for patients in hospital wards:
This algorithm is intended for use in adult patients who develop signs of symptoms of delirium during a hospital admission in an environment without continuous vital signs monitoring.

Algorithm for patients after cardiac surgery:
This algorithm is intended for use in adult patients who are admitted to the hospital for cardiac surgical procedures. The algorithm can be used in patients treated in any hospital environment, with or without continous vital signs monitoring, such as post anesthesia care, high care, medium care, step-down or high-dependency units, as well as normal wards.

Algorithm for patients in intensive care units:
Becasuse this algorithm suggests diagnostic strategies and treatments that are only appropriate in patients whose vital signs are continuously monitored, this algorithm should only be used for adult critically ill patients treated in an intensive care unit.

The International Expert Consensus Panel points out that these algorithms are intended to be used as cognitive aids by trained HCP. Each delirium or AE episode is unique, and requires urgent medical attention by a trained interdisciplinary team of HCP. Patients with delirium or AE require an individualized, patient and family oriented approach. The panel suggests that HCP follow international guidelines for the prevention, detection and treatment of delirium and AE. Differences in the management of delirium and AE may arise from the availability of resources and specific medicines, cultural and social values, population characteristics, and legislation regarding the treatment of mental disorders, in particular treatments that are applied against the patients will. The Panel strongly encourages HCP to familiarize themselves with local protocols, available resources and legislation relevant to treatment of patients with mental health disorders. The Delphi Algorithms should always we be used in respect of the aforementioned conditions.

The Algorithms are not intended for sale and may not be reproduced, printed or published without explicit approval of the International Expert Consensus Panel.

You may reach out to the panel via delirium.delphi@gmail.com

On behalf of the International Expert Consensus Panel:

Dr. Thomas H. Ottens, anesthesiologist-intensivist, chair
Prof. Dr. Arjen J.C. Slooter, neurologist-intensivist
Carsten Hermes, RN, MSc, advanced nurse practitioner

DELIRIUM DELPHI

ALGORITHMS

Algorithm for patients in HOSPITAL WARDS

This algorithm is intended for patients whose screening results indicate possible acute encephalopathy / delirium during admission to a hospital ward. All patients should receive preventive non-pharmacologic measures, regardless of their cognitive state (**see Reference Card A**).

STEP 1: VERIFY the diagnosis - consider alternative diagnoses
Perform a thorough **physical examination** for evidence of other acute, potentially life-threatening conditions that may appear similar to acute encephalopathy / delirium. See **Reference Card B** for guidance.

STEP 2: Identify and treat COMMON underlying causes
Cognitively vulnerable patients (e.g. older adults, patients with cognitive disorders) may develop delirium even from mild physiological disturbances.

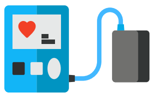

Evaluate vital signs
Screen for circulatory and respiratory insufficiency. Use an Early Warning Score system to monitor changes in vital signs over time.

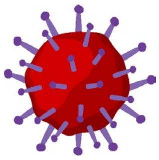

Evaluate for infection / sepsis
Screen for common infections, consider blood and other relevant cultures. Older adults and immunocompromised patients with sepsis may not have a high temperature.

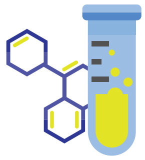

Evaluate metabolic disorders
Dehydration, electrolyte imbalance (sodium, magnesium, calcium), glucose level, metabolic acidosis, kidney or liver dysfunction.

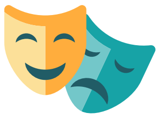

Evaluate pain, anxiety, discomfort, immobility and sleep disturbances
Consider undetected pain, bladder retention and constipation (see **Reference Card C**).
Monitor pain with behavioral scales (e.g. Behavioral Pain Scale, BPS) in non-verbal patients.

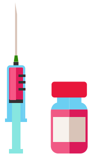

Review drugs and other intoxicants
- Evaluate all medication and possible interactions.
- Consider intoxication or withdrawal due to nicotine, alcohol and recreational drugs.
- Review sedatives and opioids: these may trigger and prolong delirium.
- Anticholinergic drugs: these may trigger and prolong delirium (see **Reference Card D**).
- Consider checking medication blood levels.

Algorithm for patients in HOSPITAL WARDS

STEP 3: Symptomatic treatment
Symptomatic treatment should be individualized, focusing on predominant signs and symptoms. Initate drug treatments only for hyperactive and psychotic features, and if non-pharmacologic measures provide insufficient relief (**Reference Card A**).

- Psychomotor agitation and anxiety: consider antipsychotics if agitation hinders nursing care or poses a safety risk. Reserve benzodiazepines as a rescue treatment for severe agitation or anxiety, as benzodiazepines may contribute to ongoing delirium. Benzodiazepines may be indicated in patients with alcohol withdrawal.

- Hallucinations and delusions: consider antipsychotics if these symptoms cause distress, anxiety or agitation.

- Somnolence, apathy and psychomotor slowing: reduce sedatives, start mobilization, physical therapy, create a stimulating environment (family visits, music, therapeutic activities).

STEP 4: MONITOR the patient's cognition and the effect of treatments
Assess frequently, using validated scales (RASS, NRS, CAM, etc), according to local guidelines.
Are the symptoms resolving with treatment of underlying causes?

YES

- Reduce symptomatic drug treatment to lowest effective dose
- Continue preventive non-pharmacologic measures (**Reference Card A**)
- Re-assess underlying causes / triggers daily
- Consider follow-up for long-term cognitive disorders

NO

STEP 5: Search for LESS COMMON underlying causes
Acute encephalopathy / delirium may persist despite optimal treatment of the underlying cause. If none of the common underlying causes is present, or delirium persists or worsens under treatment, consider less common underlying causes. These are shown in **Reference Card E**.

DELIRIUM DELPHI ALGORITHMS

Patients after Cardiac Surgery

This algorithm is intended for patients whose screening results indicate possible acute encephalopathy / delirium after undergoing cardiac surgery. All patients should receive preventive non-pharmacologic measures, regardless of their cognitive state (see **Reference Card A**).

STEP 1: VERIFY the diagnosis - consider alternative diagnoses
Perform a thorough **physical examination** for evidence of other acute, potentially life-threatening conditions that may appear similar to acute encephalopathy / delirium. See **Reference Card B** for guidance.

STEP 2: Identify and treat COMMON underlying causes
Cognitively vulnerable patients (e.g. older adults, patients with cognitive disorders) may develop delirium even from mild physiological disturbances.

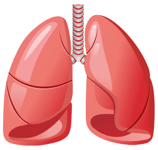

Airway & respiratory tract
Assess for airway obstruction, increased work of breathing, pain worsening with breathing, asymmetrical chest wall movement. Consider pneumothorax, pulmonary edema, pneumonia and pulmonary embolism. Check arterial blood gas. Consider chest imaging and respiratory cultures.

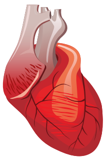

Circulation
Assess circulation by physical examination and vital signs.
Consider low cardiac output (due to arrhythmia, hypovolemia, bleeding, heart failure, tamponade)
Consider myocardial ischemia. Check ECG, Hb, lactate, cardiac ischemia markers.

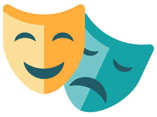

Pain, anxiety, discomfort, immobility and sleep disturbances
Consider undetected pain, bladder retention and constipation (see **Reference Card C**).
Monitor pain with behavioral scales (e.g. Behavioral Pain Scale, BPS) in non-verbal patients.

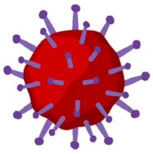

Infection / sepsis
Inspect all wounds and indwelling lines/drains. Consider endocarditis and infections of prosthetics. Measure CRP, leukocytes and/or procalcitonin, consider blood and other relevant cultures. Older adults and immunocompromised patients with sepsis may not have a high temperature.

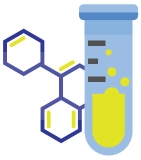

Metabolic disorders
Assess for dehydration, electrolyte imbalance (sodium, magnesium, calcium), glucose level, metabolic acidosis, kidney or liver dysfunction.

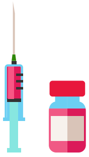

Drugs and other intoxicants
- Evaluate all medication and possible interactions.
- Consider intoxication or withdrawal due to nicotine, alcohol and recreational drugs.
- Review sedatives and opioids: these may trigger and prolong delirium.
- Anticholinergic drugs: these may trigger and prolong delirium (see **Reference Card D**).
- Consider checking medication blood levels.

Patients after Cardiac Surgery

STEP 3: Symptomatic treatment
Symptomatic treatment should be individualized, focusing on predominant signs and symptoms. Initate drug treatments only for hyperactive and psychotic features, and if non-pharmacologic measures provide insufficient relief (**Reference Card A**). The choice of symptomatic drug treatments depends on the environment.

- Psychomotor agitation and anxiety: consider **antipsychotics** if agitation hinders nursing care or poses a safety risk. *In monitored environments (ICU, HDU, PACU), consider* ***dexmedetomidine*** *or* ***clonidine***. Reserve benzodiazepines as a rescue treatment for severe agitation or anxiety, as benzodiazepines may contribute to ongoing delirium. Benzodiazepines may be indicated in patients with alcohol withdrawal.

- Hallucinations and delusions: consider **antipsychotics** if these symptoms cause distress, anxiety or agitation.

- Somnolence, apathy and psychomotor slowing: reduce sedatives, start mobilization, physical therapy, create a stimulating environment (family visits, music, therapeutic activities).

STEP 4: MONITOR the patient's cognition and the effect of treatments
Assess frequently, using validated scales (RASS, NRS, CAM, etc), according to local guidelines.
Are the symptoms resolving with treatment of underlying causes?

YES

- Reduce symptomatic drug treatment to lowest effective dose
- Continue preventive non-pharmacologic measures (**Reference Card A**)
- Re-assess underlying causes / triggers daily
- Consider follow-up for long-term cognitive disorders

NO

STEP 5: Search for LESS COMMON underlying causes
Acute encephalopathy / delirium may persist despite optimal treatment of the underlying cause. If none of the common underlying causes is present, or delirium persists or worsens under treatment, consider less common underlying causes. These are shown in **Reference Card E**.

DELIRIUM DELPHI ALGORITHMS

Patients in Intensive Care Units

This algorithm is intended for patients whose screening results indicate possible acute encephalopathy / delirium during treatment in the Intensive Care Unit. All patients should receive preventive non-pharmacologic measures, regardless of their cognitive state (**see Reference Card A**).

STEP 1: VERIFY the diagnosis - consider alternative diagnoses
Perform a thorough **physical examination** for signs of other acute, potentially life-threatening conditions that may appear similar to acute encephalopathy / delirium. See **Reference Card B** for guidance.

STEP 2: Identify and treat COMMON underlying causes
Cognitively vulnerable patients (e.g. older adults, patients with cognitive disorders) may develop delirium even from mild physiological disturbances.

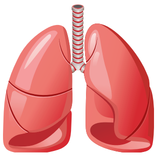

Airway & respiratory tract
Assess the respiratory tract by physical examination and monitoring parameters. Check arterial blood gas. Consider chest imaging and respiratory cultures.
Consider pneumothorax, pulmonary edema, pneumonia and pulmonary embolism.
In mechanically ventilated patients, assess and optimize ventilator settings. Consider increased work of breathing, overexertion, tube obstruction, dyssynchrony, tube discomfort, sinusitis.

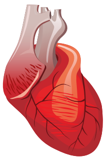

Circulatory tract
Assess circulatory system by physical examination and monitoring parameters.
Consider low cardiac output (due to arrhythmia, hypovolemia, bleeding, heart failure, tamponade)
Consider myocardial ischemia. Check ECG, Hb, lactate, cardiac ischemia markers.

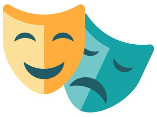

Pain, anxiety, discomfort, immobility and sleep disturbances
Consider undetected pain, bladder retention and constipation (see **Reference Card C**).
Monitor pain with behavioral scales (e.g. Behavioral Pain Scale, BPS) in non-verbal patients.

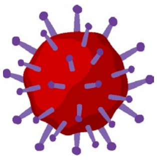

Infection / sepsis
Screen for common infections, consider blood cultures. Consider endocarditis and infections of indwelling catheters, drains or implanted prosthetics. ICU patients with sepsis may have normal or even low temperature.

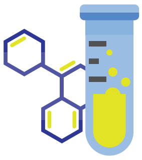

Metabolic disorders
Imbalance of sodium, ionized calcium or glucose, metabolic acidosis, kidney dysfunction (uremia) or liver dysfunction (elevated bilirubin, liver enzymes or ammonia)

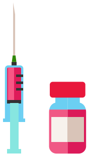

Drugs and other intoxicants
- Evaluate all medication and possible interactions.
- Consider intoxication or withdrawal due to nicotine, alcohol and recreational drugs.
- Review sedatives, hypnotics and opioids: these may trigger and prolong delirium.
- Anticholinergic drugs: these may trigger and prolong delirium (see **Reference Card D**).
- Consider checking medication blood levels.

Patients in Intensive Care Units

STEP 3: Symptomatic treatment
Symptomatic treatment should be individualized, focusing on predominant signs and symptoms. Initate drug treatments only for hyperactive and psychotic features, and if non-pharmacologic measures provide insufficient relief (**Reference Card A**). Apply physical restraints only if strictly necessary.

- Psychomotor agitation and anxiety: start with **dexmedetomidine** or **clonidine**, titrated to effect. Consider adding **antipsychotics** if agitation hinders nursing care or poses a safety risk. Reserve benzodiazepines as a rescue treatment for severe agitation or anxiety, as benzodiazepines may contribute to ongoing delirium. Benzodiazepines may be indicated in patients with alcohol withdrawal.

- Hallucinations and delusions: consider **antipsychotics** if these symptoms cause distress, anxiety or agitation.

- Somnolence, apathy and psychomotor slowing: reduce sedatives, start mobilization, physical therapy, create a stimulating environment (family visits, music, therapeutic activities).

STEP 4: MONITOR the patient's cognition and the effect of treatments
Assess frequently, using validated scales (RASS, NRS, CAM-ICU, etc), according to local guidelines.
Are the symptoms resolving with treatment of underlying causes?

YES

- Reduce symptomatic drug treatment to lowest effective dose
- Continue preventive non-pharmacologic measures (**Reference Card A**)
- Re-assess underlying causes / triggers daily
- Consider follow-up for long-term cognitive disorders

NO

STEP 5: Search for LESS COMMON underlying causes
Acute encephalopathy / delirium may persist despite optimal treatment of the underlying cause. If none of the common underlying causes is present, or delirium persists or worsens under treatment, consider less common underlying causes. These are shown in **Reference Card E**.

DELIRIUM DELPHI ALGORITHMS

Reference Card A: Nonpharmacologic Preventive Measures

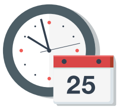

Verbally reassure the patient and facilitate reorientation. Provide a clearly visible clock and calendar. Ensure the patient has access to their glasses and hearing aids. Provide structure with a daily schedule of activity and resting hours. Involve family / loved ones in treatment.

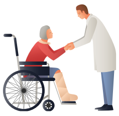

Start mobilization and physical therapy as early as possible. Provide a schedule tailored to the indiviual patient’s needs.

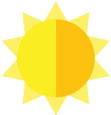

Create a stimulating environment during daytime. Expose patients to bright light during the day. Encourage listening to music and other stimulating activities. Encourage contact with loved ones. Online social contacts may be helpful to some patients.

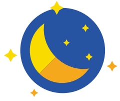

Distinguish daytime from nighttime. During nighttime, avoid medical and nursing care activities that are not strictly necessary. If preferred by the patient, provide a sleep mask and ear plugs. Limit noise and light exposure to an absolute minimum during nighttime.

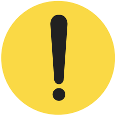

Ensure adequate hydration, nutrition and bowel care.
Avoid prolonged fasting for procedures.
Keep the use of physical restraints to an absolute minimum.
Avoid routine pre-medication for anesthesia.

Reference Card B: Alternative diagnoses in patients with possible acute encephalopathy / delirium

Perform a **physical examination** and consider the differential diagnosis.

- Focal neurological deficits: broad differential diagnosis, including **stroke and other structural brain pathology**
Consult a neurologist, obtain brain imaging

- Fever (or immune deficiency) and/or meningeal irritation: **meningitis / encephalitis**
Consult a neurologist, obtain brain imaging, lumbar puncture

- History of seizures/epilepsy or other brain disorders, abnormal eye movements, automatisms or muscle twitching: (non-convulsive) **seizures**
Consult a neurologist, obtain EEG

- Nystagmus, ocular palsy, history of undernourishment or alcohol abuse: **Wernicke encephalopathy**
Treat with high dose thiamine and consider other vitamin deficiencies

- Autonomic hyperactivity (shivering, hyperthermia, mydriasis, hypertension) with neuromuscular features (tremor, myoclonus, hyperreflexia): **serotonin syndrome**
Discontinue serotonergic drugs, consider anti-serotonergic therapy, monitor creatine kinase

- Hyperthermia, autonomic instability, rigidity, tremor, increased creatine kinase: **neuroleptic malignant syndrome** (especially after starting neuroleptic drugs)
Discontinue neuroleptic drugs, consider dopamine agonist, symptomatic treatment, monitor creatine kinase

DELIRIUM DELPHI ALGORITHMS

Reference Card C: Potential sources of pain and discomfort in non-communicating patients

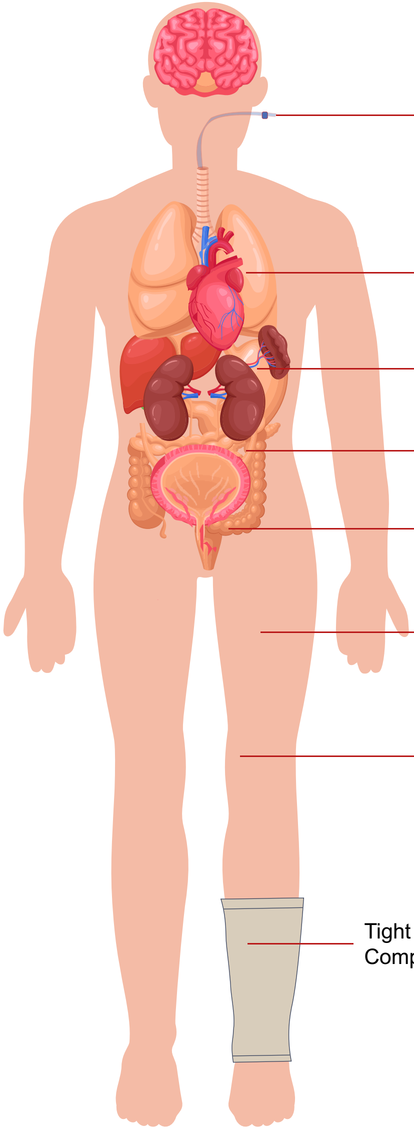

Causes	Specific signs & symptoms
Discomfort from tubes, drains and catheters	Tachypnea, ventilator dyssynchrony, oral lesions
Angina	ECG changes (ischemia, arrhythmia)
Bowel ischemia	Lactate acidosis, (bloody) diarrhoea, delayed gastric emptying
Fecal impaction	Infrequent bowel movement, delayed gastric emptying
Urinary retention	Low urine output despite normal kidney function
Decubitus	Skin damage at pressure sites, heels, occiput, and around catheters & drains
Back and leg pain	History of chronic (neuropathic) pain
Tight casts or bandages<br>Compartment syndrome	Elevated creatine kinase (CK)<br>Poor distal circulation or swelling

Reference Card D: Drugs with strong anticholinergic effects

The Anticholinergic Drug Scale (ADS) is an expert classification of anticholinergic drug effects. This is a list of commonly used drugs with anticholinergic effects. **This is not a complete list.** Less potent anticholinergic drugs may still cause relevant adverse effects, for example when combined. For patients with polypharmacy online calculation tools may be helpful to estimate total anticholinergic burden.

Anticholinergic adverse effects include dry mouth and eyes, constipation, tachycardia, urine retention and several neurocognitive effects (forgetfullness, agitation, paranoia and delirium). Risk factors for developing anticholinergic adverse effects are advanced age and dementia.

Level 3
Anticholinergic effects very likely

Amitriptyline
Atropine
Chlorpromazine
Clemastine
Clomipramine
Clozapine
Darifenacin
Desipramine
Diphenhydramine
Hydroxyzine
Hyoscyamine
Imipramine
Meclizine
Nortriptyline
Oxybutynin
Promethazine
Scopolamine
Tolterodine
Trimipramine

Level 2
Anticholinergic effects likely

Carbamazepine
Cimetidine
Cyproheptadine
Disopyramide
Meperidine
Oxcarbazepine
Pimozide
Ranitidine

DELIRIUM DELPHI ALGORITHMS

Reference Card E: Less common underlying causes of acute encephalopathy / delirium

Ensure that more common triggers, such as those mentioned in algorithm step 2, have been excluded before initiating diagnostic work-up for less common underlying causes.

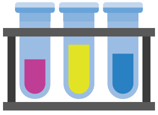

Endocrinology & deficiencies

Consider additional lab evaluation for the following conditions:

- Thyroid dysfunction
- Vitamin deficiency (B1, B12, folate)
- Hypercortisolism / adrenal insufficiency
- Hyperparathyroidism

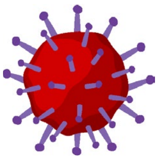

Less common infections

- Opportunistic infections
- Viral reactivation (CMV, EBV, herpes simplex)
- HIV, syphilis
- Tuberculosis

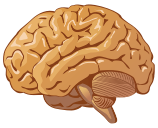

Central nervous system

Consider consulting a neurologist for the following conditions:

- Stroke
- Subdural hematoma / hygroma
- Meningitis / (auto-immune) encephalitis
- Non-convulsive seizures
- Intracranial space occupying lesions, abcesses or metastases
- Hydrocephalus
- Vasculitis
- Dementia

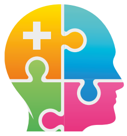

Mental health problems

Consider consulting a psychiatrist. The differential diagnosis is broad. Some psychiatric disorders may mimic delirium, have overlapping symptoms, or predispose to delirium.

- Catatonia
- Agitated depression
- Psychosis
- Mania

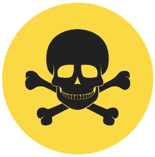

Poisoning

Consider carefully if further work-up for these causes is appropriate.

- Pesticides, solvents
- Carbon monoxide
- Mercury, manganese, lead and other (heavy) metals

List of abbreviations:

CAM: Confusion Assessment Method
CAM-ICU: Confusion Assessment Method - Intensive Care Unit version
CMV: Cytomegalovirus
ICU: Intensive Care Unit
EBV: Eppstein Barr Virus
ECG: Electrocardiogram
EEG: Electro-encephalogram
Hb: Hemoglobin
HDU: High Dependency Unit
HIV: Human Immunodeficiency Virus
NRS: Numeric Rating Scale
PACU: Post Anesthesia Care Unit
RASS: Richmond Agitation and Sedation Scale

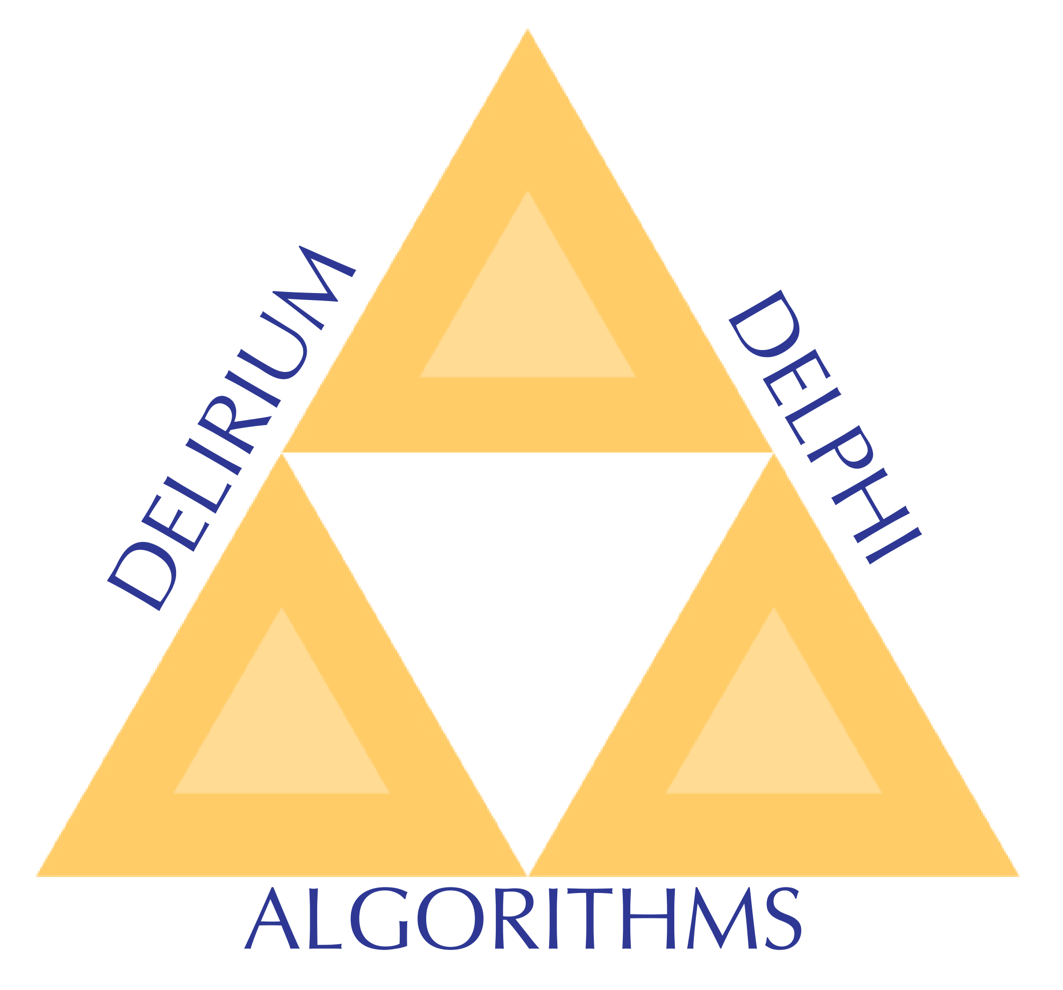

This publication contains images from Freepik.com and Flaticon.com
